# Role of Liraglutide in weight management and reproductive health in women with obesity and PCOS- A critical review of the evidence

**DOI:** 10.12688/f1000research.167998.1

**Published:** 2025-09-24

**Authors:** Shravya Shetty, Mouna Bannur Karunakara, Ravi Ram Kristipati, Guruprasad Kalthur, Sandhya Kumari

**Affiliations:** 1 Division of Reproductive Biology, Department of Reproductive Science, Kasturba Medical College, Manipal, Manipal Academy of Higher Education, Manipal, Karnataka, 576104, India; 2 Embryotoxicology Laboratory, Environmental Toxicology Group, Indian Institute of Toxicology Research- (CSIR-IITR), Lucknow, Uttar Pradesh, 226001, India; 3 Sree Mookambika Centre for Research and Innovation, Sree Mookambika Institutes, Padanilam, Kulasekharam, Tamil Nadu, 629161, India

**Keywords:** Obesity, PCOS, Insulin resistance, Hyperandrogenism, Liraglutide, Ovarian Function, Fertility

## Abstract

Obesity and polycystic ovary syndrome (PCOS) in women are associated with significant reproductive dysfunction, manifesting as hormonal imbalances, anovulation, and subfertility. These conditions not only impair reproductive outcomes but also contribute to a reduced quality of life. Crucially, obesity and PCOS share a bidirectional relationship. While obesity can trigger and worsen PCOS manifestations, the convergence of these two conditions has contributed significantly to the growing incidence of female infertility. This review critically examines liraglutide’s mechanistic role and therapeutic potential in mediating metabolic restoration and improving the fertility outcomes in women burdened by obesity and PCOS. A literature search was conducted in original databases until March 2025 in PubMed, Google Scholar, Cochrane Library, Scopus, and Web of Science using the following keywords pertinently: ‘obesity’, ‘GLP-1R’, ‘liraglutide’, ‘female reproductive system’, ‘polycystic ovarian syndrome’, ‘ovarian function’, and ‘pregnancy outcomes’. Accumulated evidence suggests that liraglutide promotes greater weight loss and improves reproductive outcomes in women with obesity and PCOS by regulating endocrine parameters, reducing systemic inflammation, restoring menstrual cyclicity, and supporting folliculogenesis. It improves reproductive outcomes by increasing natural conception rates and improving responses to assisted reproductive techniques (ART). However, with all the positive outcomes, the major limitations using this drug for weight loss are the need for daily subcutaneous injections, cost, and gastrointestinal adverse effects. Long-term assessment of the sustained efficacy and impact on reproductive outcomes, including the pattern of ovulation and offspring health, is warranted. This review consolidated present findings and emphasized areas for further exploration to better inform clinical decision-making and future research directions.

## Introduction

Obesity has expeditiously transitioned from a lifestyle-associated metabolic disorder into a global epidemic and a deeply ingrained pathological condition. It is defined by a body mass index (BMI) exceeding 35 kg/m ^2^, while overweight is defined as a BMI above 25 kg/m ^2^. ^1^ As highlighted by the National Family Health Survey (2019–2021), the demographic burden of obesity is more evident among women, with prevalence rates of abdominal obesity reaching 40% in women compared to 12% in men. ^2^ The consequences of obesity have extended far beyond being mere metabolic syndrome, with it now recognized as a significant contributor to a spectrum of multisystemic disorders, including respiratory, gastrointestinal (GI), endocrine, cardiovascular, musculoskeletal, and psychological disorders that collectively lower the quality of life. ^3^ Sharing a similar etiology with obesity, polycystic ovarian syndrome (PCOS) is the most common endocrine-metabolic disorder negatively impacting women of reproductive age, characterized by chronic anovulation, hyperandrogenism, chronic low-grade inflammation, insulin resistance, and altered hormonal profiles, including elevated luteinizing hormone (LH), reduced follicle stimulating hormone (FSH), and imbalances in estrogen and insulin levels. ^4– 6^ Around 50% of the women with PCOS are overweight or obese, and existing comorbidities of reproductive dysfunction and metabolic disturbances in both obesity and PCOS create a vicious cycle, promoting menstrual irregularities, hormonal imbalances, and subfertility. Adding to this, Infertility contributes to psychological distress, unhealthy behaviors, and additional weight gain, further compounding the cycle. ^7– 10^ Additionally, central obesity has been robustly linked to a gamut of reproductive endocrinopathies, leading to poor fertility outcomes (both assisted and spontaneous). ^11^ Therefore, early, long-term, and persistent intervention to address the condition becomes imperative. Weight loss remains a first approach and cornerstone strategy for managing both PCOS and obesity-induced infertility. Even the slightest weight loss of 5 to 6% has shown considerable benefits in restoring ovulatory cycles, increasing sex hormone-binding globulin (SHBG) levels, improving insulin sensitivity, and enhancing assisted reproductive techniques (ART) outcomes. ^12– 14^ Weight reduction by lifestyle-based interventions, such as diet restriction, physical activity is clinically effective, but is often hampered by poor adherence, limited access to resources, and socioeconomic barriers, leaving a significant gap in treatment success and sustainability. Pharmacotherapy agents, particularly liraglutide, have come up as a compelling adjunct. Liraglutide, a glucagon-like peptide-1 receptor agonist (GLP-1RA), was originally developed for glycemic control and weight management. Acting through the entero-insular axis, it enhances insulin secretion, delays gastric emptying, reduces appetite, and facilitates weight loss. ^15^ Extending therapeutic benefits beyond systemic metabolic effects, its action in improving reproductive health is better emphasized by the presence of glucagon-like peptide-1 receptors (GLP-1R) in ovarian granulosa cells, signaling at direct ovarian action. ^16^ These observations open new treatment approaches for addressing the hormonal and metabolic dysfunction mutual with PCOS, obesity, and resultant infertility.

Despite well-structured and employed public health strategies and awareness, obesity and PCOS conditions continue to prevail, with wide-reaching reproductive and endocrine implications resulting in a low quality of life. With its outstanding advantageous metabolic and reproductive outcomes, liraglutide presents a novel, practical, and non-surgical intervention in this complicated, challenging clinical space. This review aims to critically shed light on liraglutide’s mechanistic role and therapeutic potential evidenced by murine models and clinical trials, particularly its ability to mediate metabolic restoration and improve fertility outcomes in women affected by obesity and PCOS.

## Methods

A literature search was conducted in PubMed, Cochrane Library, Scopus, and Web of Science, including reviews, retrospective, cross-sectional, and prospective studies, randomized trials, systematic reviews, and meta-analyses published before May 2025. No start date was set. The search strategy included the keywords obesity, female reproductive system, PCOS, anti-obesity treatment, GLP-1RA, liraglutide, ovarian function, and pregnancy outcomes. Furthermore, older publications that are said to be very relevant are also included. Priority was given to meta-analysis, Cochrane database reviews, and randomized controlled studies for inclusion. Opinion pieces, anecdotal evidence, and articles under review are excluded.

### Effect of obesity and PCOS on reproductive function

**Bidirectional relationships of obesity and PCOS and reproductive health**

Even though obesity and PCOS are not the clinical diagnostic characteristics of each other’s conditions, the coexistence is about 30-70%. ^17^ The strong bidirectional network of metabolic, inflammatory, and endocrine disturbance between these two conditions further perpetuates the reproductive dysfunction synergistically. Obesity poses a significant risk for developing insulin resistance by triggering chronic low-grade systemic and local inflammation, which is a key diagnostic criterion for PCOS. ^18, 19^ Manifested hyperinsulinemia elevates androgen levels by reducing SHBG production in the liver and causing an elevation in ovarian androgen levels. ^20, 21^ PCOS, on the other hand, promotes visceral fat accumulation through hyperandrogenism, which is a major driver of hyperinsulinemia. ^22^ Adipose tissue acts as an endocrine organ, releasing adipokines and proinflammatory cytokines; in obese or PCOS conditions, dysregulated hypertrophy and hyperplasia of adipose tissues actively drive chronic low-grade inflammation. Together, key factors such as hyperinsulinemia, excess adipocytes, and chronic low-grade inflammation create a harmful nexus between the two conditions. ^23, 24^ The disrupted hormonal cascade interferes with steroidogenesis, impairing folliculogenesis and further worsening metabolic dysfunction that can lead to infertility in obese or PCOS women. ^25, 26^ In addition, obesity may impact fertility by many processes, such as mitochondrial dysfunction, ^27^ meiotic disruption, and excess androgen production in follicular thecal cells may result in follicular atresia and anovulation. ^11^ Therefore, reducing body weight and improving insulin sensitivity are essential for improving fertility in obese and PCOS women. Weight loss is one of the key potential therapeutic approaches to address their metabolic and reproductive challenges. Multiple clinical studies consistently reported beneficial effects, such as improvement in menstrual cyclicity, insulin resistance, and ovulation rate post-weight loss, directing a better reproductive health in these women. ^28, 29^ Studies also evidenced the improvement of metabolic, endocrine, and reproductive derangements in PCOS-affected women after successful weight loss. ^24^ Irrespective of the beneficial effects, the adherence to the weight loss programs involving lifestyle intervention only is less, and often, drop-out cases are high ^30, 31^ and lifestyle interventions alone are generally insufficient for managing long-term sustainable weight loss. ^32^ Therefore, an alternative way to achieve weight loss would be pharmacotherapy combined with lifestyle intervention. So far, in PCOS women suffering from infertility, metformin remains the drug of choice, ^33^ and research is in progress in investigating the use of other agents that target improving insulin sensitivity, such as GLP-1 RA, in treating PCOS women. In recent years, the use of anti-obesity medication has risen enormously both in obese and PCOS women. Orlistat, naltrexone-bupropion, phentermine-topiramate, liraglutide, semaglutide, setmelanotide, and trizepatide are the present Food and Drug Administration (FDA) approved anti-obesity drugs. ^34^ Although some of the drugs have shown potential in studies targeting metabolic and endocrine signaling pathways in the liver, skeletal muscle, and adipose tissue, their progress to clinical development has not yet been realized. Recent improvements in understanding the signaling pathways that regulate hunger and satiety have led to new drug developments for obesity and PCOS.

**Evolution and overview of liraglutide**

Following its approval as an anti-diabetic drug by the US FDA in 2010, liraglutide was further endorsed as an anti-obesity drug in 2014, substantiated by its robust effectiveness in both glycemic control and weight management. From laboratory discovery to market approval, liraglutide, an acylated GLP-1 analogue, it consists of 31 amino acids, shares 97% homology with endogenous GLP-1. Liraglutide has been incorporated with 2 key modifications: (1) replacing the naturally occurring lysine residue at position 34 with arginine, and (2) adding a palmitic acid moiety to the ε-amino group of the lysine residue at position 26 via a glutamic acid spacer. These modifications facilitate albumin binding, which extends the liraglutide’s half-life to over 13 hours, thereby permitting once-daily subcutaneous dosage. ^35– 37^ The multimodal action of the drug is substantiated by the GLP-1Rs found in multiple organs such as the gut, pancreas, lungs, brain, liver, kidney, adipose tissue, lungs, heart, blood vessels, reproductive system, and nervous system. ^38^ The action of the drug is aligned with the incretin hormone, whose major action is regulating food intake by delaying gastric emptying, maximizing nutrient absorption, as well as controlling the glucagon secretion and glycemic excursion post-meal. ^37^ Focusing on its brief action on the liver, pancreas, and brain in the direction of regulating blood glucose, liraglutide stimulates insulin secretion and limits hepatic glucose secretion. Extending further, it mediates effects in the brain for appetite suppression, influencing weight management. ^38^ One of its primary actions is the stimulation of insulin secretion when liraglutide binds to GLP-1R on the β-cells (beta cells) of the pancreas. The binding activates a G-protein, which subsequently elevates cyclic adenosine monophosphate (cAMP) levels and activate protein kinase A (PKA), initiating a signaling cascade that leads opening of voltage-gated calcium channels and increases intracellular calcium concentration, which triggers the fusion of insulin-containing vesicles with the plasma membrane, thereby promoting insulin secretion. ^39^ Anti obese effects are mainly driven centrally by activating GLP-1 receptors in the brain stem as well as hypothalamic nuclei. Liraglutide enhances satiety by regulating reward pathways and vagal afferent signaling. It inhibits NPY (Neuropeptide Y) /AgRP (Agouti-Related Protein) neurons (reducing hunger) and stimulates POMC (Pro-Opiomelanocortin) /CART (Cocaine- and Amphetamine-Regulated Transcript) neurons promoting satiety, thereby increasing the post-meal period, and reducing energy intake. In long-term weight loss maintenance, liraglutide primarily drives sustainability by preserving leptin levels and increasing PYY (peptide YY), leading to an adaptive metabolic slowdown. ^40^

In addition, its effect on appetite suppression and weight management, liraglutide demonstrates a wide range of protective effects across various tissues and cell types, including central aspects of reproductive health such as the hypothalamic-pituitary-gonadal (HPG) axis and ovarian cells. These benefits are not restricted to a single target or organ but arise through interconnecting signaling pathways. Its mechanisms involve modulation of key regulators such as AMP-activated protein kinase (AMPK), phosphoinositide 3-kinase (PI3K)/protein kinase B (Akt), Wingless/integrated site (Wnt)/β-catenin, and autophagy-related pathways, allowing it to influence inflammation, oxidative stress, apoptosis, metabolism, and tissue remodeling in diverse biological contexts. ^37– 40^ Most importantly, these pathways actively participate in exerting effects on reproductive tissues as well. By targeting the central signaling cascades, liraglutide not only addresses metabolic outcomes but also supports the improvement in reproductive health.

### Mechanisms and clinical utility of liraglutide in managing metabolic dysregulation

**Effect of liraglutide on weight loss and insulin sensitivity**

Liraglutide has been extensively investigated across diverse populations and pathological conditions, particularly for its anti-diabetic, anti-obesity, and metabolic benefits. Table 1 summarizes the key findings of liraglutide in weight loss in obese and diabetic patients. Clinical trials have demonstrated a dose-dependent weight loss of 4.8 kg, 5.5 kg, 6.3 kg, and 7.2 kg in subjects receiving liraglutide for 20 weeks at doses of 1.2 mg, 1.8 mg, 2.4 mg, and 3.0 mg, respectively compared to a weight loss of 2.8 kg with placebo and 4.1 kg with orlistat, a lipase inhibitor (120 mg, three times daily, orally), alongside an energy-deficient diet and physical activity. ^41^ However, the occurrence of adverse effects such as nausea and vomiting was more frequent in liraglutide with increasing dose compared to placebo and orlistat, and these GI adverse effects were transient. Participants taking orlistat also reported a higher frequency of GI events as compared to those on placebo and reported a higher frequency of diarrhea than those treated with liraglutide. No events of acute pancreatitis were reported after liraglutide treatment, a known adverse effect commonly associated with other GLP-1RAs. Consistent with its weight reducing effect, treatment with liraglutide (3 mg daily) for 56 weeks resulted in higher body weight reduction (8.4 ± 7.3 kg) compared to placebo group (2.8 ± 6.5 kg), even when participants in both groups received counseling for lifestyle modifications, underscoring the independent efficacy of the drug. It reduced fasting glucose, glycated hemoglobin (HbA1c), and fasting insulin levels, and was associated with improved physical and mental health, thereby improved the healthy quality of life as compared to placebo. ^42^ Continued long-term studies have further pronounced its beneficial effects in obese women as treatment with 2.4 mg daily during 1 ^st^ year, followed by 3.0 mg daily in 2 ^nd^ year, resulted in superior weight loss (7.8 kg) compared to placebo (2.0 kg) and orlistat (3.9 kg), indicating its well tolerability, sustained weight loss and greater efficacy with improved metabolic and cardiovascular risk factors over orlistat. ^43^ Beyond weight management and improvements in glycemic control, liraglutide has demonstrated additional metabolic benefits such as improvements in lipid profile and hepatic enzyme parameters, along with body mass index reduction in type 2 diabetes (T2D) patients. ^44^ It has also been shown to reduce visceral fat, improve insulin sensitivity and β cell function in metformin-treated obese patients with early or prediabetes as compared to obese individuals who were undergoing lifestyle modifications. ^45^ Treatment with liraglutide for 12 months in obese and prediabetic women achieved a significant reduction in visceral fat as well as insulin sensitivity with comparable weight loss (7%) to lifestyle counseling. It enhanced insulin sensitivity as early as two weeks of treatment before achieving significant weight loss. ^46^ These benefits were mediated via GLP receptor activation, as the effect was nullified by the GLP-1R antagonist exendin. Weight loss has also been a crucial factor in improving cardiovascular health, extending to renal improvement in metabolically compromised individuals. Liraglutide significantly reduced major adverse cardiovascular events, cardiovascular mortality, and nephropathy risk in T2D patients with high cardiovascular risk ^47– 49^ (Supplementary Table 1). Cardioprotective effects of it were supported by a cohort with prior myocardial infarction or stroke ^48^ with reductions in both vascular events and nephropathy. ^49^ In addition, liraglutide has been demonstrated renal protection, with a randomized controlled trial showing delayed onset and progression of diabetic kidney disease compared to placebo in T2D patients. ^50^

**Table 1.** Summarizes key clinical studies demonstrating the anti-obesity efficiency of liraglutide.

Author	Indicators	Concentration of liraglutide	Effect of liraglutide as an anti-obese drug
Astrup et al. ^41^ double-blind, placebo-controlled trial	Obese women	1.2, 1.8, 2.4, or 3.0 mg daily with lifestyle modifications for 20 weeks	Weight loss was associated with reductions in waist circumference, systolic and diastolic blood pressure, as well as a decreased prevalence of metabolic syndrome and prediabetes.
Astrup et al. ^42^ placebo-controlled trial	76% women with stable body weight, BMI ≥ 30 kg/m ^2^ and ≤ 40 kg/m ^2^	Continued with ^40^ dosage was 2.4 mg daily for 20-70 weeks and 3.0 mg daily for 70-96 weeks	Superior weight loss was observed in the liraglutide group at both end points (year 1 and year 2) compared to placebo and orlistat. Decreased systolic blood pressure was accompanied by unchanged pulse rate. Mean FPG and HbA _1c_ levels were reduced, as well as improved lipid profiles.
Inoue et al. ^43^ observational study	T2D women	0.3-0.9 mg/day based on the tolerance for the drug for 2 years	Long-term treatment improved glycemic control, improved lipid profile, and maintained body weight reduction.
Pi-Sunyer et al. ^44^ double-blind, randomized controlled trial	Obese women	Scaled from 0.6 mg daily to 3 mg daily with lifestyle modification for 56 weeks	When used as an adjuvant to increased physical activity and a reduced-calorie diet, it led to greater reductions in body weight, glycated hemoglobin, fasting glucose, and fasting insulin levels, along with improvements in insulin resistance and β-cell function.
Santilli et al. ^45^ random trial longitudinal, randomized, controlled, parallel-arm study	Obese T2D subjects	Scaled from 0.6 to 1.8 mg daily for 3–12 months	Even with the comparable weight loss in both the liraglutide and lifestyle intervention arms, pronounced visceral fat loss, and improvement in β-cell function were observed in the liraglutide arm.
Mashayekhi et al. ^46^ double-blind and placebo-controlled study	Obese men and women	1.8 mg/day liraglutide & 100 mg/day sitagliptin. The treatment period was 2 weeks and 14 weeks	Liraglutide improved the HOMA-IR index even without significant weight loss, whereas the sitagliptin and hypocaloric diet group had no change.

BMI: Body Mass Index; FPG: Fasting Plasma Glucose; HbA1c: Haemoglobin A1c; HOMA-IR: Homeostatic Model Assessment of Insulin Resistance; T2D: Type 2 Diabetes.

**Figure 1.**
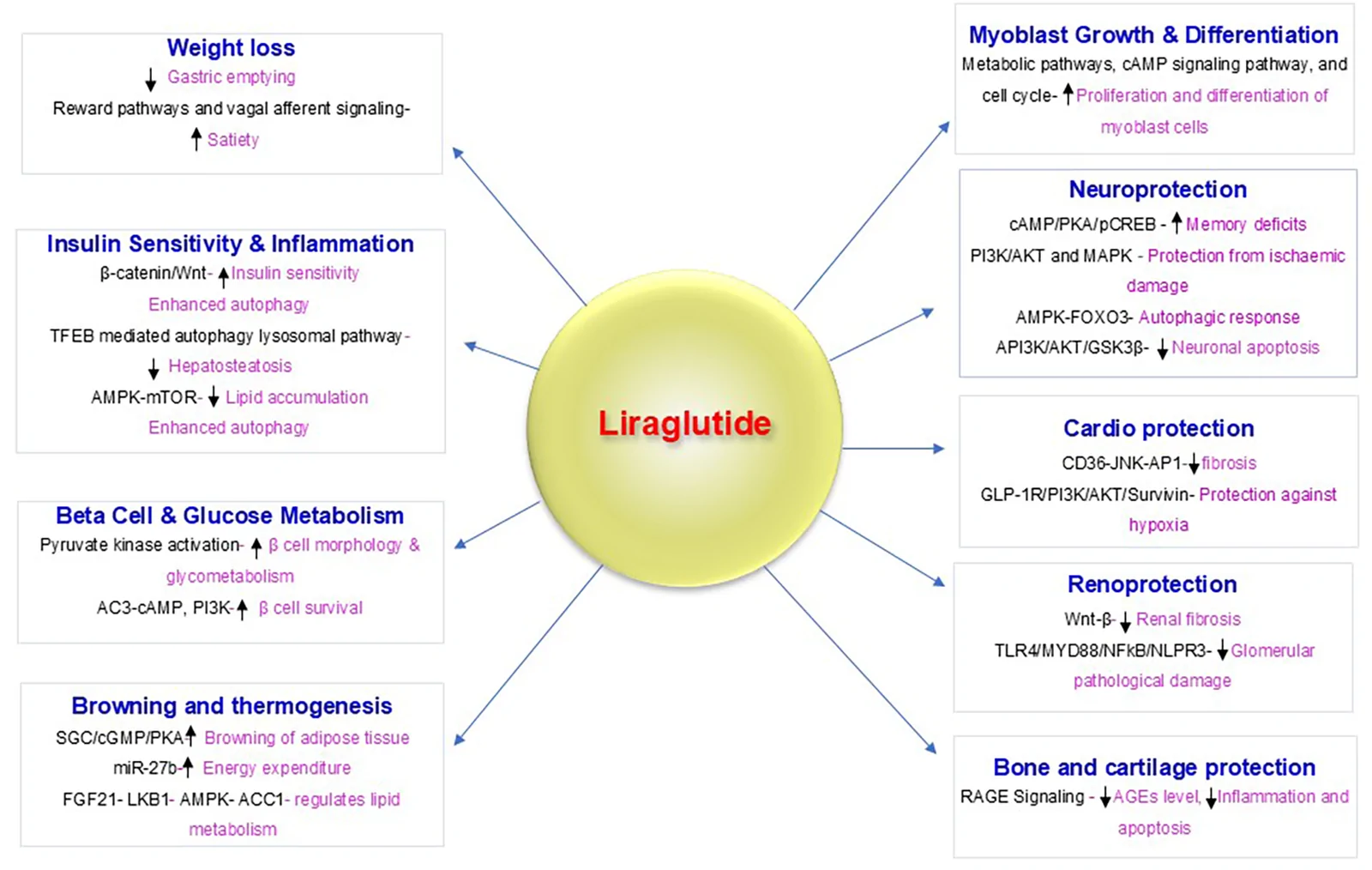
This schematic illustrates the diverse signaling pathway by which liraglutide modulates autophagy, fibrosis, inflammation, apoptosis, lipid accumulation, and cellular morphology & metabolism, exerting the protective effect across multiple organ systems. AC3: Adenylate Cyclase 3; AMPK: AMP-Activated Protein Kinase; AMPK-FOXO3: AMP-Activated Protein Kinase - Forkhead Box O3; AMPK-mTOR: AMP-Activated Protein Kinase - Mechanistic Target of Rapamycin; API3K: Phosphoinositide 3-Kinase; AP1: Activator Protein 1; cAMP: Cyclic Adenosine Monophosphate; cAMP/PKA/pCREB: Cyclic Adenosine Monophosphate/Protein Kinase A/phosphorylated cAMP Response Element-Binding Protein; CD36: Cluster of Differentiation 36; FGF21: Fibroblast Growth Factor 21; GSK3β: Glycogen Synthase Kinase 3 Beta; GLP-1R: Glucagon-Like Peptide-1 Receptor; JNK: c-Jun N-terminal Kinase; LKB1: Liver Kinase B1; MAPK: Mitogen-Activated Protein Kinase; miR-27b: microRNA-27b; mTOR: Mechanistic Target of Rapamycin; MYD88: Myeloid Differentiation Primary Response 88; NFkB: Nuclear Factor kappa-light-chain-enhancer of activated B cells; NLPR3: NOD-, LRR- and pyrin domain-containing protein 3; PKA: Protein Kinase A; PI3K: Phosphoinositide 3-Kinase; pCREB: phosphorylated cAMP Response Element-Binding Protein; RAGE: Receptor for Advanced Glycation End-products; SGC: Soluble Guanylate Cyclase; Survinin: Baculoviral IAP Repeat-Containing Protein 5; TFEB: Transcription Factor EB; TLR4: Toll-Like Receptor 4; Wnt: Wingless/Integrated; β-catenin: Beta-catenin.

Delving into the mechanisms involved in these beneficial clinical outcomes, liraglutide’s actions are exerted by modulating several key pathways as summarized in Figure 1, and Supplementary Table 1 details the liraglutide-mediated mechanistic orchestra. One strategic way liraglutide drives anti-obesity effects is through modulation of fat metabolism and reduction of insulin resistance. Liraglutide upregulates brown fat differentiation and thereby promotes thermogenesis and mitochondrial biogenesis in brown adipose tissue (BAT) through soluble guanylyl cyclase (sGC)/cyclic guanosine phosphate (cGMP)/PKA pathway signaling, further complementing its anti-obesity effects by increasing energy expenditure ^51^ and the promotion of heat production (thermogenesis) through miR-27b-related mechanisms. ^52^ It promoted the browning of white fat by downregulating the expression of miR-27b, which in turn increased the levels of browning-associated proteins. The connection between fat metabolism and the liver is demonstrated *in vivo* using obese mouse models, where liraglutide upregulates adenylate cyclase 3 (AC3) ^53^ at both hepatic and serum levels, hinting at a coordinated response to improve energy balance and metabolic health.

Improved insulin sensitivity is achieved independent of weight loss seen in liraglutide treatment, as AC3 expression was not correlated with insulin levels. ^45^ Improved insulin sensitivity in the diabetic mice model after liraglutide treatment was achieved by inhibiting gluconeogenic enzymes such as glucose-6-phosphatase and phosphoenolpyruvate carboxykinase through activation of the Wnt/β-catenin signaling pathway. ^54^ Simultaneously, it enhances the expression of glucose transporter type 4 (GLUT4) in both liver and skeletal muscle. ^55^ It enhanced insulin secretion in pancreatic β-cells, and glycolysis and glycogenesis via activation of hexokinase and pyruvate kinase enzymes in the liver. It preserves β-cell mass by inhibiting apoptosis through cAMP-PI3K signaling pathway ^56^ and increases the proliferation rate by reducing oxidative stress in diabetic condition *in vitro.* ^57^ At the cellular level, liraglutide is closely tied to its ability to stimulate autophagy via transcription Factor EB (TFEB), the master regulator of autophagy activation, which promotes lysosomal biogenesis and clearance of lipid droplets, thus attenuating hepatic lipid accumulation. ^58^ Autophagy also plays a preventive role in lipotoxic liver injury, where liraglutide enhances autophagy and protects against hepatocyte damage via activating the AMPK pathway. ^59^ These effects were also observed in adipose tissue, where liraglutide activates the fibroblast growth factor 21 (FGF21)/liver kinase B1(LKB1)/AMPK/acetyl-CoA carboxylase 1 (ACC1) axis, thereby reducing lipogenesis, promoting fatty acid oxidation, and suppressing pro-inflammatory signaling. ^60^ Crucially, all these tissue-specific effects converge to increase systemic insulin sensitivity. Improved insulin sensitivity facilitates the reduction of tau hyperphosphorylation and β-amyloid deposition, a hallmark in Alzheimer’s disease models. Liraglutide, by improving insulin sensitivity and decreasing β-site amyloid precursor protein cleaving enzyme 1 (BACE1) activity, the enzyme responsible for initiating β-amyloid production, exhibits a neuroprotection effect. ^61^

Another crucial factor for the exacerbation of insulin resistance & obesity is leptin resistance. Leptin has a beneficial effect on glucose-insulin metabolism, decreasing the degree of insulin resistance and hyperinsulinemia, ^62^ it also promotes ovarian thecal cells and granulosa cells’ functions directly through AK/STAT and MAPK signaling pathways. ^63^ However, in an obese condition, often impaired leptin signaling subverts the benefits and prolongs metabolic dysfunction. Higher leptin levels, generally observed in PCOS conditions, inhibit follicular growth as well as hinder oocyte maturation. ^64^ Liraglutide, through a dual mechanism, in obese mice combats impaired leptin signaling by (i), improves leptin sensitivity by enhancing phosphorylation of STAT3 (signal transducer and activator of transcription 3), a key mediator of leptin function, through the Janus kinase 2/signal transducer and activator of transcription 3 (JAK2-STAT3) signaling pathway (ii) lowers the expression of inhibitory regulators like the protein tyrosine phosphatase 1B (PTP1B) and suppressor of cytokine signaling 3 (SOCS3) of leptin signaling pathway. ^65^ Comprehensive metabolic improvements, such as enhanced insulin sensitivity, restored leptin signaling, and visceral fat reduction observed after the liraglutide treatment, and thus it creates a favorable environment relevant to improving reproductive health. Insulin resistance, for example, leads to consequent disruption of the menstrual cycle through the elevation of androgen levels, through activation of adrenocorticotropic hormones. ^66^ Liraglutide improves insulin resistance, lowers fasting insulin levels, decreases hyperandrogenism, and possibly thereby improves menstrual cyclicity pattern. ^67, 68^ Thus it establishes a favorable metabolic milieu through multiple pathways, possibly thereby improving ovarian health. However, further research is warranted to learn the direct method of action and long-term consequences on ovarian function.

**Effect of liraglutide on modulation of inflammatory responses**

Liraglutide demonstrates strong anti-inflammatory activity across diverse tissue cells, establishing groundwork for the broader therapeutic utility (Supplementary Table 1). In human chondrocytes, it inhibits the nuclear factor κB (NF-κB) signaling pathway and blocks tumor necrosis factor-alpha (TNF-α)-induced degradation of extracellular matrix (ECM) proteins, thereby protecting against arthritis. It also inhibits advanced glycation end products (AGE)-induced inflammation by reducing both matrix degradation and chondrocyte apoptosis via blocking receptor for advanced glycation end products (RAGE) signaling. ^69^ Similarly, in fibroblasts exposed to high-glucose conditions, liraglutide modulates the cluster of differentiation 36 (CD36)/c-Jun N-terminal kinase (JNK)/activator protein 1 (AP1) pathway, leading to downregulation of prolyl 4-hydroxylase subunit alpha 1 (P4HA1) enzyme, a key enzyme involved in collagen synthesis. This modulation results in reduced collagen overproduction, and subsequently attenuates cell proliferation, invasion, migration, and fibrotic changes. ^70^ These effects extend to renal fibrosis, where liraglutide suppresses Wnt/β-catenin signaling to reduce extracellular matrix deposition and improve renal function in mesangial cells exposed to high glucose conditions that mimic diabetic nephropathy. ^71^ Liraglutide downregulates the Toll-Like Receptor 4 (TLR4)/myeloid differentiation primary response 88 (MyD88)/NF-κB inflammatory cascade, which suppresses the expression of pro-inflammatory cytokines and reduces renal inflammation and fibrosis in diabetic nephropathy. ^72^ These pathways are further supported, where it reduces glomerular sclerosis, attenuates albuminuria and histological renal damage in early-stage diabetic kidney disease by activating autophagy in renal cells. ^73^ In parallel, vascular and neuroprotection also roots from its anti-inflammatory effects. In addition, it reduced carotid intima-media thickness and inflammatory markers in overweight individuals with impaired glucose tolerance. ^74^ Further expanding the beneficial effects regarding bone metabolism, it increased bone formation markers, enhanced bone mineral density at the lumbar spine and femoral neck, and decreased bone resorption, suggesting a protective role against osteoporosis in diabetic patients. ^75^ Neuroprotective effects have also been reported, with improved brain activation and cognitive function in T2D patients after treatment with liraglutide. ^76^ In neuronal cell cultures and animal models, it displays consistent neuroprotective effects. In models of mild traumatic brain injury, it enhances neurotrophic support by improving cell survival and supports neural repair via cAMP/PKA/cAMP response element-binding protein (CREB) pathway. ^77^ This pathway plays a crucial role in promoting the expression of neurotrophic factors, which are essential for neuronal growth, synaptic plasticity, and long-term neuronal survival. Through this mechanism, it not only reduces neuronal damage but also promotes a favorable environment for brain recovery following injury. The PI3K/Akt and Mitogen-Activated Protein Kinase (MAPK) pathways are central to neuroprotective effects in ischemic injury, where it prevents neuronal apoptosis. ^78^ It also promotes neurogenesis, promotes neurite outgrowth, and activates the Wnt signaling pathway in response to oxidative stress in cortical neurons. ^79^ Further, activation of AMPK/FOXO3 (Forkhead Box O3) signaling, it enhances autophagy and reduces neuronal damage in spinal cord injury, further inhibits neuronal apoptosis and ultimately reduces tissue damage. It supports cellular energy balance and stress resistance, thereby contributing to functional recovery after spinal cord injury. ^80^ Additionally, it protects against neonatal hypoxic-ischemic brain injury by activating the PI3K/Akt/glycogen synthase kinase 3 β (GSK3β) signaling and reduces oxidative stress and inflammatory responses, thereby preserving brain tissue and improving neurological outcomes. ^81^ It reverses synaptic loss, memory impairment, and improves cognition in transgenic models of Alzheimer’s disease, ^82, 83^ and in Huntington’s disease, improves motor coordination and neuronal viability. ^84^

Chronic low-grade inflammation is a major factor in the interconnected pathophysiology of PCOS and obesity is chronic low-grade inflammation which affects ovarian function and leads to compromised ovarian growth and ovarian failure. ^85^ In PCOS condition, follicular fluid and granulosa cells express elevated levels of C-X-C motif chemokine ligand 10 (CXCL10), which is one of the major mediators pro proinflammatory conditions in the ovary. The anti-inflammatory profile of liraglutide is very relevant and it lowers the level of CXCL10 by blocking the JAK signaling pathway in PCOS condition. ^86^ It also exhibits potent anti-inflammatory effects by inhibiting the NF-kB pathway, followed by upregulation of Sirtuin 1 (SIRT1) and downregulation of TNF-α an obese diabetic condition. ^87^ The decrease in TNF-α levels depicts improved insulin resistance, as TNF-α is a known mediator of insulin resistance. It mediates insulin resistance by phosphorylating serine of insulin receptor substrate (IRS-1) in insulin-sensitive tissues and decreasing expression of GLUT 4, an insulin-sensitive glucose transport protein. ^88^ Furthermore, the beneficial effect of liraglutide on decreased inflammation is by lowering key proinflammatory markers, including Interleukin-6 (IL-6), Interleukin-1beta (IL-1β), and chemokine C-C motif ligand 2 (CCl2). ^89, 90^

Chronic low-grade inflammation is closely connected to reactive oxygen species (ROS). The delicate balance between ROS and the ovarian antioxidant defense system is crucial for maintaining cellular homeostasis, and an imbalance typically leads to ovarian disorders, such as ovarian endometriosis and ovarian cancer. ^91^ However, complexity arises about the relationship between liraglutide and oxidative stress. Although liraglutide exerts potent antioxidant effects in stress conditions, ^92, 93^ it has been shown to increase oxidative stress, and induce granulosa cell apoptosis, leading to follicular atresia in the ovarian tissues in non-disease condition. ^94^ Most importantly, this adverse effect of liraglutide observed was reversed post-discontinuation of the drug and can be attributed to the dose and model of the study. Overall, liraglutide highlights the anti-inflammatory effects at both systemic and organ levels, balancing the inflammatory milieu. However, it also warrants further investigation into its effects on ovarian oxidative stress and granulosa cell survival to optimize the use of the drug in correcting reproductive dysfunction in obesity and PCOS conditions.

**Role of liraglutide on modulation of gut microbiota**

A balanced gut microbial profile emphasizes hormone regulation and metabolic health. ^95^ In obese and PCOS conditions, imbalance of gut microbiota is associated with a higher degree of hyperandrogenism and elevated insulin resistance. An abundant amount of bacteria-derived butyrate produced by members of the Bacillota phylum, specifically *Lachnospiraceae* and *Oscillospiraceae*, has been shown to have beneficial effects on immune response regulation, insulin sensitivity, and intestinal homeostasis, all of which are dysregulated in obesity and metabolic disturbance conditions. Metabolites like short-chain fatty acids (SCFAs) produced by the gut microbiota are also known to positively influence systemic inflammation and insulin sensitivity, exhibiting anti-obese, anti-inflammatory properties, two vital aspects of female reproductive health. ^96– 98^ Liraglutide alters the overall diversity and abundance of gut microbiota, elevates well known probiotics namely, Bifidobacterium and Ileibacterium, promotes bacteria-derived butyrate production and improves lipid profile, inducing weight reduction, regulates glucose metabolism, and reduces insulin resistance and hyperandrogenism, ultimately promoting better metabolic health in obese and PCOS, and in diabetic condition. ^99– 102^ Its effect is mediated by enrichment of the phylum Bacillota, leading to a reduced Bacilliota to Bacteroidota ratio, which was lower in the PCOS condition. ^103– 105^ Liraglutide medication has been linked in clinical trials to better ovarian morphology and function, as seen by increased monthly regularity and decreased ovarian volume. ^68^ These improvements are likely mediated through modulating gut microbiota, which in turn influences hormonal balance and systemic inflammation. However, further clinical trials are warranted to confirm the therapeutic effects of liraglutide.

### Effect of liraglutide on improving reproductive health

**Effect of liraglutide on modulating reproductive hormones and follicular development**

**Figure 2.**
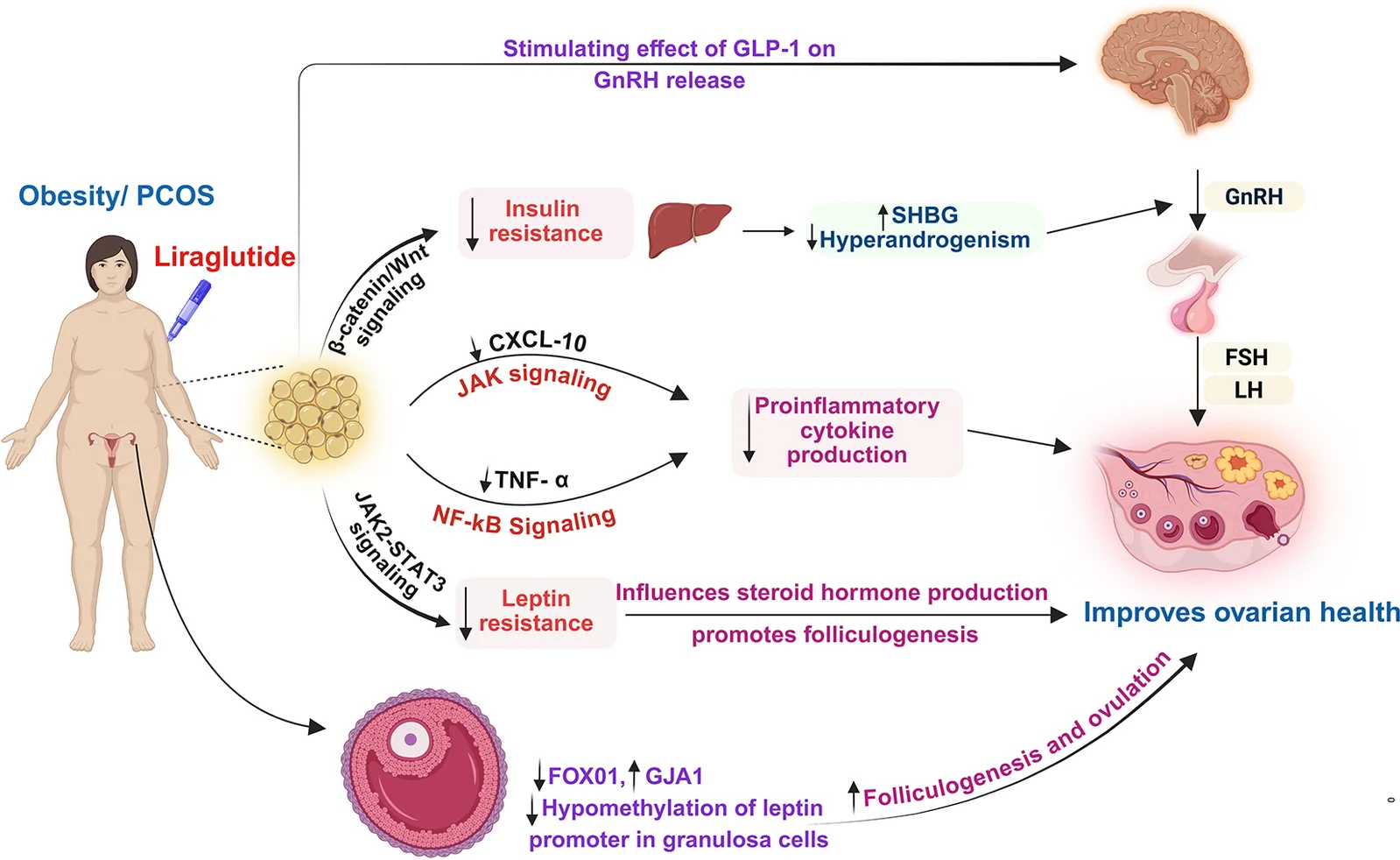
Effect of liraglutide in improving ovarian health explains the mechanism of liraglutide in improving folliculogenesis through various mechanisms (created with BioRender.com). It regulates GnRH pulsatile, FSH and LH level through HPO axis, reduces proinflammatory cytokines, and regulates steroid hormone production thereby affecting ovarian function. At follicle level, It also improves GJA1 protein homeostasis between oocytes and granulosa cells, supporting the effective cellular communication, improves granulosa cell survival by reducing FOXO1 expression, reduces the proinflammatory cytokines in the follicular fluid, reduces the methylation of the leptin promoter in granulosa cells, promoting the improvement in follicular development and ovulation, aiding the improved natural pregnancy rates. (Black upward arrow: increases; black downward arrow: decreases;). CXCL10: C-X-C motif chemokine ligand 10; FSH: Follicle stimulating hormone; FoxO1: Forkhead box protein O1; GJA1: Gap junction protein 1; GnRH: Gonadotropin-releasing hormone; GLP-1: Glucagon-like peptide-1; HPO: Hypothalamus-pituitary-ovary; JAK2: Janus kinase 2; LH: Luteinizing hormone; NF-kB: Nuclear factor kappa-light-chain-enhancer of activated B cells; PCOS: Polycystic ovary syndrome; SHBG: sex hormone-binding globulin; STAT3: signal transducer and activator of transcription 3; TNF-α: Tumor Necrosis Factor alpha; Wnt/β-catenin signaling: Wingless-related integration site/beta-catenin signaling.

One of the promising strategies of liraglutide in improving the reproductive profile is hormone profile modification by interconnected metabolic and endocrine modifications ( Figure 2). Hyperinsulinemia and elevated LH secretion in obese and PCOS are the key mediators of ovarian-associated hyperandrogenism. Substantial weight loss reduces adipose tissue inflammation and lipotoxicity and thereby decreases hyperinsulinemia and improves insulin sensitivity, ^106^ which in turn reduces androgen synthesis by following the PI3K pathway mediated CYP17 driven androgen synthesis activity. ^107^ Improved insulin resistance elevates SHBG (sex hormone binding globulin), compounding the lowered androgen levels, resulting in lowered free androgen index. ^108^ Liraglutide has exhibited an increase in SHBG levels and a decrease in total testosterone, free androgen index. ^68, 109, 110^ This is a noteworthy change, as both are linked to the degree of hirsutism. ^111^ Further, anti-Mullerian hormone (AMH) produced by granulosa cells stimulates the pulsatile release of LH and FSH by gonadotropin-releasing hormone (GnRH) is closely associated with protecting ovarian reserve and function, observed to be elevated in the obese or PCOS condition. ^112, 113^ Marking all the positive alterations in terms of improving the hormonal index and menstrual cyclicity, liraglutide may be a useful treatment choice for women undergoing PCOS & obesity, characterizing hormonal imbalances.

Recent studies have underlined several specific mechanisms through which liraglutide improves ovarian health by supporting follicular development ( Figure 2). Liraglutide reduced methylation of the LEP (leptin) promoter in ovarian granulosa cells in PCOS conditions, ^114^ which positively connected to higher ovulation rates, better hormonal profiles, and spontaneous conception. It alters the phosphorylation status of forkhead box protein O1 (FoxO1), a known negative regulator of cell survival, by promoting Akt-mediated phosphorylation, resulting in reduced expression of pro-apoptotic genes such as Bim and FasL, resulting in decreased apoptosis of granulosa cells and improved folliculogenesis. ^115^ The administration of Liraglutide restores the equilibrium between granulosa cells and oocytes by improving the expression of the gap junction protein alpha 1 (GJA1) in PCOS conditions through the JAK) signaling pathway. ^86^ Importantly, it upregulated Ferredoxin 1 (FDX1) expression in PCOS condition, a protein critical for p450 enzyme activation, which is suppressed in this condition. ^116^ It also reversed PCOS-induced downregulation of steroidogenic enzymes namely cholesterol side-chain cleavage enzyme (CYP11A1) and aromatase (CYP19A1) and impaired follicular development. The restoration of these enzymes led to improved estradiol synthesis, folliculogenesis and overall reproductive health. These beneficial effects liraglutide are further driven by its potential anti-inflammatory effect, which favorably enables the ovarian microenvironment for promoting folliculogenesis and ovulation. The dual function of liraglutide, in increasing granulosa cell survival and reducing inflammation, makes it a potential therapeutic option for improving follicular growth in PCOS conditions.

**Clinical trials assessing the effect of liraglutide on metabolic, hormonal, and reproductive function in obese and PCOS women**

Liraglutide has demonstrated promising effects in improving metabolic and reproductive parameters in nondiabetic women with obesity and PCOS ( Table 2). A randomized, placebo-controlled phase 3 clinical trial involving 82 obese and PCOS patients reported a significant weight reduction in the liraglutide group (5.7%), compared to the placebo group (1.4%). ^67^ The treatment also led to notable reductions in free androgen index, improved hyperandrogenism, and cardiometabolic profiles, along with more regular menstrual cyclicity, and two participants achieved spontaneous conception. An 18% dropout rate was observed, primarily due to the patients’ lack of adherence to treatment and the occurrence of pregnancy, and in the placebo group, due to no weight loss. The major adverse events reported during intervention were nausea, vomiting, headache, diarrhea, constipation, and injection site reactions. The other limitations of the trial included its short trial duration, lack of ovulation or live birth assessment, and absence of clinical evaluation of hirsutism. Effects like lowered testosterone and improved menstrual cyclicity were pronounced in an extreme case of PCOS with HAIR-AN syndrome, an endocrine disorder depicted by Insulin resistance, hyperandrogenism and Acanthosis Nigricans. Administration of liraglutide in women with HAIR-AN syndrome led to improvements in insulin resistance, improved lipid profile and adiposity, with one participant achieving pregnancy and delivering a healthy child. ^68^ Notably, SHBG was reduced post-liraglutide treatment 14 ± 4 months in these patients. Interestingly, these benefits occurred with minimal weight loss, underscoring its pharmacologic effects independent of weight reduction. In a study involving 72 women with overweight and PCOS, liraglutide led to reduction in body weight, 19% increase in SHBG level was supported by the decrease in free testosterone level (19%). However, it did not improve insulin sensitivity, although reductions in fasting glucose and leptin levels were observed. This study did not elaborate on reproductive functions as well as hormone levels. ^109^ Furthermore, a 16-week treatment of liraglutide versus metformin in a cohort of 30 obese PCOS patients, revealing liraglutide was superior in improving glucose metabolism, lipid profiles, and BMI, as well as reducing leptin promoter methylation in granulosa cells compared to metformin treatment and compared to before the therapy started. ^110^ Increasing the leptin secretion from granulosa cells may be able to treat the symptoms of patients with obesity or PCOS. Moreover, significant reductions in FSH, LH, and E2 levels were observed after liraglutide intervention, alongside improved menstrual regularity, ovulation rates, and natural pregnancy occurrences. However, this study lacked a placebo group, had a small sample size, and did not evaluate proinflammatory cytokines. In a randomized trial, obese women with PCOS treated with liraglutide (1.6 mg/day) for 12 weeks resulted in the greatest significant weight loss, followed by roflumilast and metformin, while metformin showed the least among the three treatment groups. The roflumilast, a Phosphodiesterase 4 (PDE4) inhibitor, increases intracellular cAMP level and regulates lipid metabolism and modulates inflammation, while metformin, an AMPK activator, regulates hepatic glucose production and improves insulin sensitivity and is commonly used in the treatment of PCOS. All three treatment groups experienced reductions in total testosterone and free androgen index, with improvements in menstrual regularity, but roflumilast had a significant upper hand over the liraglutide arm, though no significant intergroup differences in free testosterone, SHBG, androstenedione, dehydroepiandrosterone (DHEA), FSH, and LH were observed. Treatment with liraglutide resulted in improved glucose homeostasis compared to metformin and roflumilast subjects. However, this study had a few limitations, including a small sample size, short duration, open-label nature of the study, and lack of active lifestyle promotion. ^117^ In contrast, a double blind, placebo-controlled, randomized trial involving 65 overweight PCOS women treated with liraglutide (1.8 mg/day) for 26 weeks observed lowered free testosterone levels, an increase in the levels of SHBG, while the testosterone level was unaltered. This study also observed improved fasting glucose and HbA1c levels, indicating reduced insulin resistance were also noted. The treated patients showed average weight loss of 5.2 kg, improved menstrual regularity, and reduced ovarian volume by 1.6 mL. The improvement in menstrual bleeding pattern was attributed to both substantial weight loss and improved insulin sensitivity. The reduction in body weight may have also led to normalization of AMH level. Three-dimensional ultrasound revealed a decrease in both total ovarian volume and stromal volume. However, this study also had a limitation of participant selection bias, as partial recruitment was by social media, and lifestyle changes were not actively promoted throughout the study. ^118^ Among the Chinese population, the combination therapy of metformin and liraglutide has proved its efficacy in weight loss and improving hyperandrogenemia, including total testosterone, free androgen index, and SHBG levels. After 12 weeks of treatment, combination group showed a menstrual cycle recovery rate of 92.59%, whereas the metformin group showed 88% recovery rate. ^110^ Nevertheless, limitations included the absence of a control group, potential selection bias, lack of fertility outcome data, short follow-up duration, and their small sample size, single-center design. In addition, this study did not assess key reproductive parameters such as ovulation or pregnancy outcomes.

**Table 2.** Clinical evidence of liraglutide alone or in combination with metformin improving in reproductive, metabolic, and endocrine dysfunction in obese and PCOS women.

Author, study & design	Population	Dosage & treatment duration of liraglutide	Effect of liraglutide on metabolic, endocrine, reproductive changes
Elkind-Hirsch et al. ^67^ Double-blind RCT	Obese, PCOS women	Liraglutide 3 mg/day vs. placebo + lifestyle, for 32 weeks.	Decreased weight, and increased insulin sensitivity. Improved endocrine and metabolic markers over placebo. More regular menstrual cyclicity and out of 44 patients, 2 pregnancies, healthy births.
Livadas et al. ^68^ Case Study	HAIR-AN syndrome/PCOS women	Liraglutide 1.8 mg/day, for 14 months.	Decrease of insulin resistance, androgen levels, with improvement of fat deposition. More regular menstrual cyclicity and out of 5 patients, 1 pregnancy, live birth.
Jensterle et al. ^117^ Prospective RCT	Obese, PCOS women	Liraglutide 1.2 mg QD, s.c. vs. metformin 1000 mg BID or roflumilast 500 μg, QD, 12 weeks.	No significant change in hyperandrogenism. Increased menstrual frequency outperformed metformin in reproductive parameters.
Nylander et al. ^118^ Double-blind RCT	Overweight PCOS women	Liraglutide 1.8 mg vs. placebo, 26 weeks follow-up.	Effective weight loss, decreased fasting glucose, hemoglobin A1c, increased SHBG, and decreased free testosterone. Decreased ovarian and stromal volume, and more regular menstrual cyclicity.
Xing et al. ^110^ Prospective, Open-label RCT	PCOS women	Metformin 1000 mg BID or liraglutide 1.2 mg QD, s.c. + metformin for 12 weeks.	In combination with metformin, it improved bodyweight, fasting glucose, hyperandrogenism, and sex hormone balance. Recovery rate of the menstrual cycle was better than metformin group in the combination group.
Jensterle et al. ^121^ Open-label RCT	Obese, PCOS women	Liraglutide 1.2 mg QD vs. metformin + liraglutide for 12 weeks.	In combination with metformin Increased glucose metabolism, decreased BMI, decreased androstenedione, and greater improvements in ovarian morphology and metabolism.
Long et al. ^122^ Retrospective	Obese, PCOS women	Liraglutide 0.6 mg QD + metformin 0.85 g BID for 12 weeks.	In combination with metformin, it improved lipid metabolism, insulin sensitivity, decreased free androgen index better then when metformin given alone. Reproductive functions not assessed.
Salamun et al. ^123^	Prospective RCT Infertile, obese PCOS women	Metformin1000 mg BID vs. metformin + liraglutide 1.2 mg QD for 12 weeks.	In combination with metformin, it decreased fasting glucose, OGTT, HOMA-IR, decreased LH & testosterone, improved metabolic and endocrine outcomes. Increased pregnancy rate per embryo transfer (85.7% in combination with metformin).

BID: Bis in die (twice daily); BMI: Body Mass Index; HAIR-AN: Hyperandrogenism, Insulin Resistance, and Acanthosis Nigricans; HbA1c: Hemoglobin A1c; HOMA-IR: Homeostatic Model Assessment of Insulin Resistance; LH: Luteinizing Hormone; OGTT: Oral Glucose Tolerance Test; PCOS: Polycystic Ovary Syndrome; QD: Quaque die (once daily); RCT: Randomized Controlled Trial; s.c.: Subcutaneous; SHBG: Sex Hormone-Binding Globulin.

Adding on, the combination of liraglutide with metformin appears to offer synergistic benefits on metabolic and reproductive outcomes in obese women with PCOS. It provides greater body weight reduction when combined with metformin compared to metformin alone and lifestyle modification. Compelling evidence suggests that the combination of liraglutide and metformin not only offers superior glycemic control and regulates HbA1c levels more effectively but also provides a significant advantage in promoting body weight loss, emphasizing its dual role in metabolic regulation in comparison to placebo, sitagliptin, glimepiride, dulaglutide, insulin glargine, and NPH insulin. ^119, 120^ When PCOS obese women who had shown a poor response to metformin treatment previously were treated with liraglutide in combination with metformin for 3 months, it led to a significant reduction in body weight. Importantly, the combined treatment did not increase the risk of hypoglycemia but induced a higher incidence of adverse effects of gastrointestinal disturbances, such as nausea, compared to metformin. However, the severity of these adverse effects was reduced over time. ^121^ A study in the Chinese population in combination therapy of metformin and liraglutide has proved its efficacy again in weight loss and improving hyperandrogenemia, including total testosterone, free androgen index, and SHBG levels. Approximately 88.24% achieved at least 5% weight loss. Nevertheless, limitations included the absence of a control group, potential selection bias, lack of fertility outcome data, short follow-up duration and their small sample size, single-center design. ^122^ This study did not assess key reproductive parameters such as ovulation, menstrual cyclicity, or pregnancy outcomes. Subsequently conducted randomized 12-week trial involving 28 infertile obese PCOS patients treated either with metformin alone or in combination with low-dose liraglutide, resulting in markedly higher pregnancy rates per embryo transfer in the combination group (85.7% vs. 28.6%), and greater cumulative pregnancy rates (69.2% vs. 35.7%), despite similar reductions in weight and visceral adiposity. ^123^ Higher cumulative pregnancy rates are supported by improved menstrual cycle regularity, glucose metabolism, and anthropometric indices. Combination therapy showed superior reductions in hyperandrogenemia (evidenced by improved SHBG and reductions in total testosterone and free androgen index) and better modulation of LH and progesterone levels. These findings suggest liraglutide is effective for weight reduction and regulated hormonal balance in women with obesity and PCOS, which supports its potential as a therapeutic option that may exert beneficial effects on reproductive outcomes in women with metabolic disorders, providing a potential therapeutic option for addressing infertility in this population.

## Discussion

Liraglutide, the foremost GLP-1 R agonist, has emerged as pivotal in the dual management of obesity and diabetes. It seeks more attention due to its rise in global consumption, potentially enabling a more holistic and comprehensive treatment approach. Notably, along with clinical triumphs of successful weight loss and adipokine rebalancing, liraglutide reshapes its therapeutic horizon, directing towards synced treatment for reproductive dysfunction. Liraglutide has demonstrated its multifaceted effects in the PCOS murine model, indicating the rescue of folliculogenesis, ^86^ improvement of granulosa cell proliferation, ^114^ which are crucial for balanced steroidogenesis, and alleviation of a proinflammatory environment. Crucially, clinical trials have reinforced the effectiveness of the drug, which significantly improved both pregnancy rates in combination with metformin and successful embryo transfer. ^124^ Modulation of gut microbiota sufficiently enriching *Lachnospiraceae* contributes to the reproductive benefits as well. ^101^ Endometrium receptivity is another crucial aspect of fertility, hampered during the conditions of obesity and PCOS ^125^ and possibly reversed by GLP-1 R agonist. ^126^ However, daily subcutaneous injection poses a challenge as it may lead to injection site reactions and pain sensations, which are often reported. ^127^ A promising alternative to daily subcutaneous injection is transdermal delivery. Liraglutide encapsulated tannic acid and aluminum ion nanoparticles have proved durable and painless with long-term therapeutic efficacy. Efforts in this direction have further led to exploring the oral route of intake. Prolonged release of liraglutide by chitosan nanoparticles, i.e., 34% within 102 hours, and protection of liraglutide from degradation by gastric and intestinal fluid has shed a ray of hope. ^128^ The right mode of administration also reduces the side effects, like gastrointestinal reactions, traditionally observed in liraglutide dosage. ^129^ Despite the advancement, factors like patient dropout due to side effects and unaffordable cost ^130^ remain significant barriers. Further studies are required in the direction of providing an affordable generic version of liraglutide, evaluating the long-term durability of the reproductive benefit, and identifying biomarkers that could also pave the way for personalized therapy in improving fertility, as well as tailored dosage.

## Conclusion

Liraglutide is a crucial addition to the treatment toolkit for women with PCOS-related obesity and reproductive dysfunction. Evidence from randomized controlled trials and retrospective studies highlights the efficacy of liraglutide in modulating hormone levels and improving insulin sensitivity, which contributes to regular menstrual patterns. Notably, few studies have further investigated liraglutide’s direct impact on reproductive success by assessing and demonstrating increased natural pregnancy rates and responses to ART. Despite these successes and advancements, critical limitations to consider include brief intervention durations, small sample sizes, lack of ovulation tracking, and limited long-term follow-up. Future studies should focus on elucidating the biochemical processes underlying liraglutide’s long-term effects on reproductive health and its potential role in more comprehensive fertility therapies. By embracing cutting-edge treatments like liraglutide, we can better address the complex challenges of infertility and empower women on their journey to reproductive health. Additionally, further research in this area will enhance the lives of women facing these challenges and lead to improved clinical outcomes. The findings underscore the importance of individualized treatment strategies in addressing the intricate issues of PCOS and obesity-related infertility.

## Ethical approval

Ethical approval and consent were not required.

## Data Availability

The data availability statement for this study has been duly registered and archived in the Biostudies repository, which is recognized for its commitment to the accessibility and preservation of scientific data. The extended data ( **Supplementary Table 1)** supported by this study is publicly available and can be accessed at the following DOI link: https://doi.org/10.6019/S-BSST2135. ^131^ Data are available under the terms of the CC0 1.0 Universal.
