# Role of Liraglutide in weight management and reproductive health in women with obesity and PCOS- A critical review of the evidence

**DOI:** 10.12688/f1000research.167998.2

**Published:** 2026-04-24

**Authors:** Shravya Shetty, Mouna Bannur Karunakara, Ravi Ram Kristipati, Guruprasad Kalthur, Sandhya Kumari

**Affiliations:** 1 Division of Reproductive Biology, Department of Reproductive Science, Kasturba Medical College, Manipal Academy of Higher Education, Manipal, Karnataka, 576104, India; 2 Embryotoxicology Laboratory, Environmental Toxicology Group, Indian Institute of Toxicology Research- (CSIR-IITR), Lucknow, Uttar Pradesh, 226001, India; 3 Sree Mookambika Centre for Research and Innovation, Sree Mookambika Institutes, Padanilam, Kulasekharam, Tamil Nadu, 629161, India

**Keywords:** Obesity, PCOS, Insulin resistance, Hyperandrogenism, Liraglutide, Ovarian Function, Fertility

## Abstract

Obesity and polycystic ovary syndrome (PCOS) in women are associated with significant reproductive dysfunction, manifesting as hormonal imbalances, anovulation, and subfertility. These conditions not only impair reproductive outcomes but also contribute to a reduced quality of life. Crucially, obesity and PCOS share a bidirectional relationship. While obesity can trigger and worsen PCOS manifestations, the convergence of these two conditions has contributed significantly to the growing incidence of female infertility. This review critically examines liraglutide’s mechanistic role and therapeutic potential in mediating metabolic restoration and improving the fertility outcomes in women burdened by obesity and PCOS. A literature search was conducted in original databases from inception until March 2025 in PubMed, Google Scholar, Cochrane Library, Scopus, and Web of Science using the following keywords pertinently: “GLP-1R”, “liraglutide”, “female reproductive system”, “polycystic ovarian syndrome”, “ovarian function”, and “pregnancy outcomes”. Accumulated evidence suggests that liraglutide promotes greater weight loss and improves reproductive outcomes in women with obesity and PCOS by regulating endocrine parameters, reducing systemic inflammation, restoring menstrual cyclicity, and supporting folliculogenesis. It improves reproductive outcomes by increasing natural conception rates and improving responses to assisted reproductive techniques (ART). However, with all the positive outcomes, the major limitations of using this drug for weight loss are the need for daily subcutaneous injections, cost, and gastrointestinal adverse effects. Long-term assessment of the sustained efficacy and impact on reproductive outcomes, including the pattern of ovulation and offspring health, is warranted. This review consolidated present findings and emphasized areas for further exploration to better inform clinical decision-making and future research directions.

## Introduction

Obesity has expeditiously transitioned from a lifestyle-associated metabolic disorder into a global epidemic and a deeply ingrained pathological condition. It is defined by a body mass index (BMI) exceeding 35 kg/m ^2^, while overweight is defined as a BMI above 25 kg/m ^2^. ^1^ As highlighted by the National Family Health Survey (2019–2021), the demographic burden of obesity in India is more evident among women, with prevalence rates of abdominal obesity reaching 40% in women compared to 12% in men. ^2^ The consequences of obesity have extended far beyond being mere metabolic syndrome, with it now recognized as a significant contributor to a spectrum of multisystemic disorders, including respiratory, gastrointestinal (GI), endocrine, cardiovascular, musculoskeletal, and psychological disorders that collectively lower the quality of life. ^3^ Sharing a similar etiology with obesity, polycystic ovarian syndrome (PCOS) is the most common endocrine-metabolic disorder negatively impacting women of reproductive age, characterized by chronic anovulation, hyperandrogenism, chronic low-grade inflammation, insulin resistance, and altered hormonal profiles, including elevated luteinizing hormone (LH), reduced follicle stimulating hormone (FSH), and imbalances in estrogen and insulin levels. ^4– 6^ Around 50% of the women with PCOS are overweight or obese, and existing comorbidities of reproductive dysfunction and metabolic disturbances in both obesity and PCOS create a vicious cycle, promoting menstrual irregularities, hormonal imbalances, and subfertility. Adding to this, Infertility contributes to psychological distress, unhealthy behaviors, and additional weight gain, further compounding the cycle. ^7– 10^ Additionally, central obesity has been robustly linked to a gamut of reproductive endocrinopathies, leading to poor fertility outcomes (both assisted and spontaneous). ^11^ Therefore, early, long-term, and persistent intervention to address the condition becomes imperative. Weight loss remains a first approach and cornerstone strategy for managing both PCOS and obesity-induced infertility. Even the slightest weight loss of 5 to 6% has shown considerable benefits in restoring ovulatory cycles, increasing sex hormone-binding globulin (SHBG) levels, improving insulin sensitivity, and enhancing assisted reproductive techniques (ART) outcomes. ^12– 14^ Weight reduction by lifestyle-based interventions, such as diet restriction, and physical activity, is clinically effective, but is often hampered by poor adherence, limited access to resources, and socioeconomic barriers, leaving a significant gap in treatment success and sustainability. Pharmacotherapy agents, particularly liraglutide, have come up as a compelling adjunct. Liraglutide, a glucagon-like peptide-1 receptor agonist (GLP-1RA), was originally developed for glycemic control and weight management. Acting through the entero-insular axis, it enhances insulin secretion, delays gastric emptying, reduces appetite, and facilitates weight loss. ^15^ Extending therapeutic benefits beyond systemic metabolic effects, its action in improving reproductive health is better emphasized by the presence of glucagon-like peptide-1 receptors (GLP-1R) in ovarian granulosa cells, signalling at direct ovarian action. ^16^ These observations open new treatment approaches for addressing the hormonal and metabolic dysfunction mutual with PCOS, obesity, and resultant infertility.

Despite well-structured and employed public health strategies and awareness, obesity and PCOS conditions continue to prevail, with wide-reaching reproductive and endocrine implications resulting in a low quality of life. With its outstanding advantageous metabolic and reproductive outcomes, liraglutide presents a novel, practical, and non-surgical intervention in this complicated and, challenging clinical space. This review aims to critically shed light on liraglutide’s mechanistic role and therapeutic potential evidenced by murine models and clinical trials, particularly its ability to mediate metabolic restoration and improve fertility outcomes in women affected by obesity and PCOS.

## Methods

**Figure 1.**
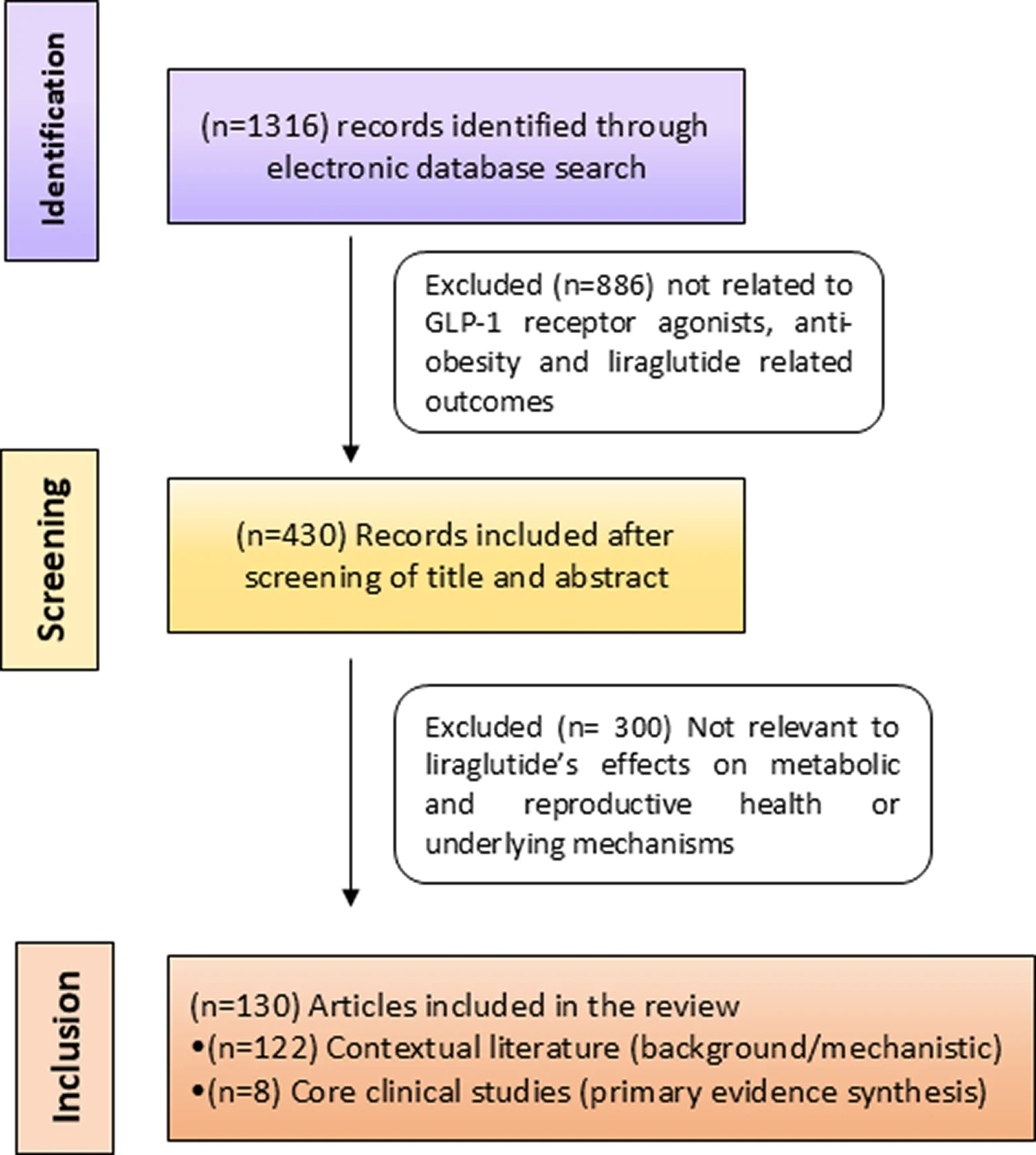
Flow diagram illustrating the study selection process for the evidence synthesis. Records were identified through database searching, screened based on title and abstract, and assessed for eligibility. Studies were excluded due to lack of relevance to GLP-1 receptor agonists, liraglutide-related metabolic, reproductive, and mechanistic outcomes. A total of 130 articles were included, comprising 122 contextual (background/mechanistic) studies and 8 core clinical studies.

A comprehensive literature search was conducted across four databases: PubMed, Cochrane Library, Scopus, and Web of Science, covering articles published through inception to March 2025. This review adopts a narrative synthesis approach to critically evaluate the mechanistic role and therapeutic potential of liraglutide in managing metabolic and reproductive dysfunction in women with obesity and PCOS. The search strategy employed a combination of keywords: “obesity”, “female reproductive system”, “PCOS”, “anti-obesity treatment”, “GLP-1RA”, “liraglutide”, “ovarian function”, and “pregnancy outcomes”. To ensure reliability and to minimise bias, two independent researchers screened title, abstract and full-text articles. The literature search strategy is illustrated in detail in Figure 1. Inclusion criteria focused on relevant articles that provided insights into mechanistic and clinical trials evaluating liraglutide’s efficacy. Non-English articles, opinion pieces, and anecdotal evidence were excluded. Of the 130 articles identified through database searches, a significant portion is categorised as contextual literature to provide historical background and support the discussion of biological mechanisms. To maintain a rigorous translational focus, the review also consists of eight clinical studies providing human evidence that establishes the real-world efficacy of liraglutide in managing the reproductive-metabolic intersection of obesity and PCOS.

### Bidirectional relationship of obesity and PCOS and reproductive health

Even though obesity and PCOS are not the clinical diagnostic characteristics of each other’s conditions, the coexistence is about 30–70%. ^17^ The strong bidirectional network of metabolic, inflammatory, and endocrine disturbance between these two conditions further perpetuates the reproductive dysfunction synergistically. Obesity poses a significant risk for developing insulin resistance by triggering chronic low-grade systemic and local inflammation, which is a key diagnostic criterion for PCOS. ^18^ ^,^ ^19^ Manifested hyperinsulinemia elevates androgen levels by regulating hepatic SHBG production and raising ovarian androgen levels. ^20^ ^,^ ^21^ Meanwhile, PCOS promotes visceral fat accumulation through hyperandrogenism, a major driver of hyperinsulinemia. Further, obese or PCOS conditions, dysregulated hypertrophy and hyperplasia of adipose tissues actively drive chronic low-grade inflammation. ^22^ Collectively, these factors disrupt hormonal cascade, interfering with steroidogenesis, impairing folliculogenesis, and further worsening metabolic dysfunction, ultimately contributing to infertility in women with obesity and/or PCOS. ^23^ ^–^ ^26^ In addition, obesity may impact fertility by many processes, such as mitochondrial dysfunction, ^27^ meiotic disruption, and excess androgen production in follicular thecal cells, which may result in follicular atresia and anovulation. ^11^ Therefore, reducing body weight and improving insulin sensitivity are the key potential therapeutic approaches to address their metabolic, endocrine and reproductive health in these women. Multiple clinical studies consistently reported beneficial effects, such as improvement in menstrual cyclicity, insulin resistance, and ovulation rate post-weight loss, directing a better reproductive health in these women. ^28^ ^,^ ^29^ Irrespective of the beneficial effects, the adherence to the weight loss programs involving only lifestyle intervention is less, and often, dropout cases are high. ^30^ ^,^ ^31^ Further, lifestyle interventions alone are generally insufficient for managing long-term sustainable weight loss. ^32^ Therefore, pharmacotherapy combined with lifestyle intervention represents an alternative strategy to achieve weight loss. In women with obese and PCOS suffering from infertility, metformin remains the drug of choice, ^33^ while ongoing research is exploring the therapeutic potential of other insulin-sensitising agents such as roflumilast, dulaglutide, exenatide, liraglutide, lixisenatide and semaglutide in the management of PCOS. In recent years, the use of anti-obesity medication has risen enormously among obese and PCOS women. Orlistat, naltrexone-bupropion, phentermine-topiramate, liraglutide, semaglutide, setmelanotide, and trizepatide are the present Food and Drug Administration (FDA) approved anti-obesity drugs. ^34^ Advances in understanding the neuroendocrine pathways regulating hunger and satiety have further accelerated the development of these therapies, with GLP-1 receptor agonists gaining increasing attention.

Following its approval as an anti-diabetic drug by the US FDA in 2010, liraglutide was further endorsed as an anti-obesity drug in 2014, substantiated by its robust effectiveness in both glycaemic control and weight management. It is an acylated GLP-1 analogue, consisting of 31 amino acids and sharing 97% homology with endogenous GLP-1, with a half-life ranging from 11 to 15 hours, enabling once-daily subcutaneous dosing. ^35^ ^–^ ^38^ The primary action of the drug is regulating food intake by delaying gastric emptying, enhancing satiety, and regulating insulin and glucagon secretion from beta cells (β-cells) of the pancreas, while reducing hepatic glucose secretion, thereby contributing to weight management. ^37^ These functions are mediated by activating a G-protein, which subsequently elevates cyclic adenosine monophosphate (cAMP) levels and activates protein kinase A (PKA), initiating a signalling cascade, that leads to the opening of voltage-gated calcium channels and increases intracellular calcium levels, which further triggers the fusion of insulin-containing vesicles with the plasma membrane and promoting insulin secretion. ^39^ In long-term weight loss maintenance, it primarily drives sustainability by preserving leptin levels and increasing peptide YY (PYY), leading to an adaptive metabolic slowdown. ^40^

In addition to its effect on appetite suppression and weight management, it demonstrates a wide range of protective effects across various tissues and cell types, including central aspects of reproductive health such as the hypothalamic-pituitary-gonadal (HPG) axis and ovarian cells. These benefits are not restricted to a single target or organ but arise through interconnecting signalling pathways. Its mechanisms involve modulation of key regulators such as AMP-activated protein kinase (AMPK), phosphoinositide 3-kinase (PI3K)/protein kinase B (Akt), wingless/integrated site (Wnt)/β-catenin, and autophagy-related pathways, allowing it to influence inflammation, oxidative stress, apoptosis, metabolism, and tissue remodelling in diverse biological contexts. ^37^ ^–^ ^40^ By targeting the central signalling cascades, liraglutide not only addresses metabolic outcomes but may also support reproductive function. ^37^

### Clinical utility of liraglutide in managing metabolic dysregulation

Liraglutide has been extensively investigated for its anti-diabetic, anti-obesity, and metabolic benefits. As summarised in Table 1, clinical trials have demonstrated dose-dependent weight loss in obese and diabetic patients following subcutaneous administration. A dose-dependent weight loss was observed in subjects receiving liraglutide for 20 weeks at various doses, with a maximum reduction of 7.2 kg achieved at 3.0 mg, respectively, which notably outperforms the 2.8 kg observed in placebo and 4.1 kg with orlistat, a lipase inhibitor (120 mg, three times daily, orally), alongside an energy-deficient diet and physical activity. ^41^ While these results are promising, transient GI effects were more frequent in both liraglutide and orlistat compared to placebo. However, liraglutide was associated with a lower frequency of diarrhoea than orlistat and no events of acute pancreatitis were noted. The efficacy of the drug is further validated by a treatment with liraglutide (3 mg daily) for 56 weeks, which resulted in nearly triple weight reduction (8.4 ± 7.3 kg) compared to the placebo group (2.8 ± 6.5 kg), despite both groups receiving counselling for lifestyle modifications, underscoring the independent efficacy of the drug. It reduced fasting glucose, glycated haemoglobin (HbA1c), and fasting insulin levels and was associated with improved physical and mental health, thereby improving the healthy quality of life as compared to placebo. ^42^ Further long-term studies have demonstrated its beneficial effects in obese women, where 2.4 mg daily during year 1, followed by 3 mg daily in year 2, resulted in superior weight loss (7.8 kg) compared to placebo (2.0 kg) and orlistat (3.9 kg), indicating its better tolerability and sustained weight loss. ^43^ Beyond weight management and improvements in glycemic control, 12 months of liraglutide treatment has been shown to reduce visceral adiposity, improve insulin sensitivity and β cell function in metformin-treated obese patients with prediabetes compared to lifestyle modifications alone, with comparable weight loss of 7%. ^44^ ^–^ ^46^ Surprisingly, in these subjects, improved insulin sensitivity was observed as early as two weeks of treatment before achieving significant weight loss. Liraglutide significantly reduced major adverse cardiovascular events, cardiovascular mortality, and nephropathy risk in T2D patients with high cardiovascular risk. ^47^ ^–^ ^50^ (Supplementary Table 1).

**Table 1.** Summarizes key clinical studies demonstrating the anti-obesity efficiency of liraglutide.

Author	Indicators	Concentration of liraglutide	Effect of liraglutide as an anti-obese drug
Astrup et al. ^41^ double-blind, placebo-controlled trial	Obese women	1.2, 1.8, 2.4, or 3.0 mg daily with lifestyle modifications for 20 weeks	Weight loss was associated with reductions in waist circumference, systolic and diastolic blood pressure, as well as a decreased prevalence of metabolic syndrome and prediabetes.
Astrup et al. ^42^ placebo-controlled trial	76% women with stable body weight, BMI ≥ 30 kg/m ^2^ and ≤ 40 kg/m ^2^	Continued with ^40^ dosage was 2.4 mg daily for 20-70 weeks and 3.0 mg daily for 70-96 weeks	Superior weight loss was observed in the liraglutide group at both end points (year 1 and year 2) compared to placebo and orlistat. Decreased systolic blood pressure was accompanied by unchanged pulse rate. Mean FPG and HbA _1c_ levels were reduced, as well as improved lipid profiles.
Inoue et al. ^43^ observational study	T2D women	0.3-0.9 mg/day based on the tolerance for the drug for 2 years	Long-term treatment improved glycemic control, improved lipid profile, and maintained body weight reduction.
Pi-Sunyer et al. ^44^ double-blind, randomized controlled trial	Obese women	Scaled from 0.6 mg daily to 3 mg daily with lifestyle modification for 56 weeks	When used as an adjuvant to increased physical activity and a reduced-calorie diet, it led to greater reductions in body weight, glycated hemoglobin, fasting glucose, and fasting insulin levels, along with improvements in insulin resistance and β-cell function.
Santilli et al. ^45^ random trial longitudinal, randomized, controlled, parallel-arm study	Obese T2D subjects	Scaled from 0.6 to 1.8 mg daily for 3–12 months	Even with the comparable weight loss in both the liraglutide and lifestyle intervention arms, pronounced visceral fat loss, and improvement in β-cell function were observed in the liraglutide arm.
Mashayekhi et al. ^46^ double-blind and placebo-controlled study	Obese men and women	1.8 mg/day liraglutide & 100 mg/day sitagliptin. The treatment period was 2 weeks and 14 weeks	Liraglutide improved the HOMA-IR index even without significant weight loss, whereas the sitagliptin and hypocaloric diet group had no change.

BMI: Body Mass Index; FPG: Fasting Plasma Glucose; HbA1c: Haemoglobin A1c; HOMA-IR: Homeostatic Model Assessment of Insulin Resistance; T2D: Type 2 Diabetes.

### Mechanisms of liraglutide involved in managing metabolic and reproductive dysfunction

**Figure 2.**
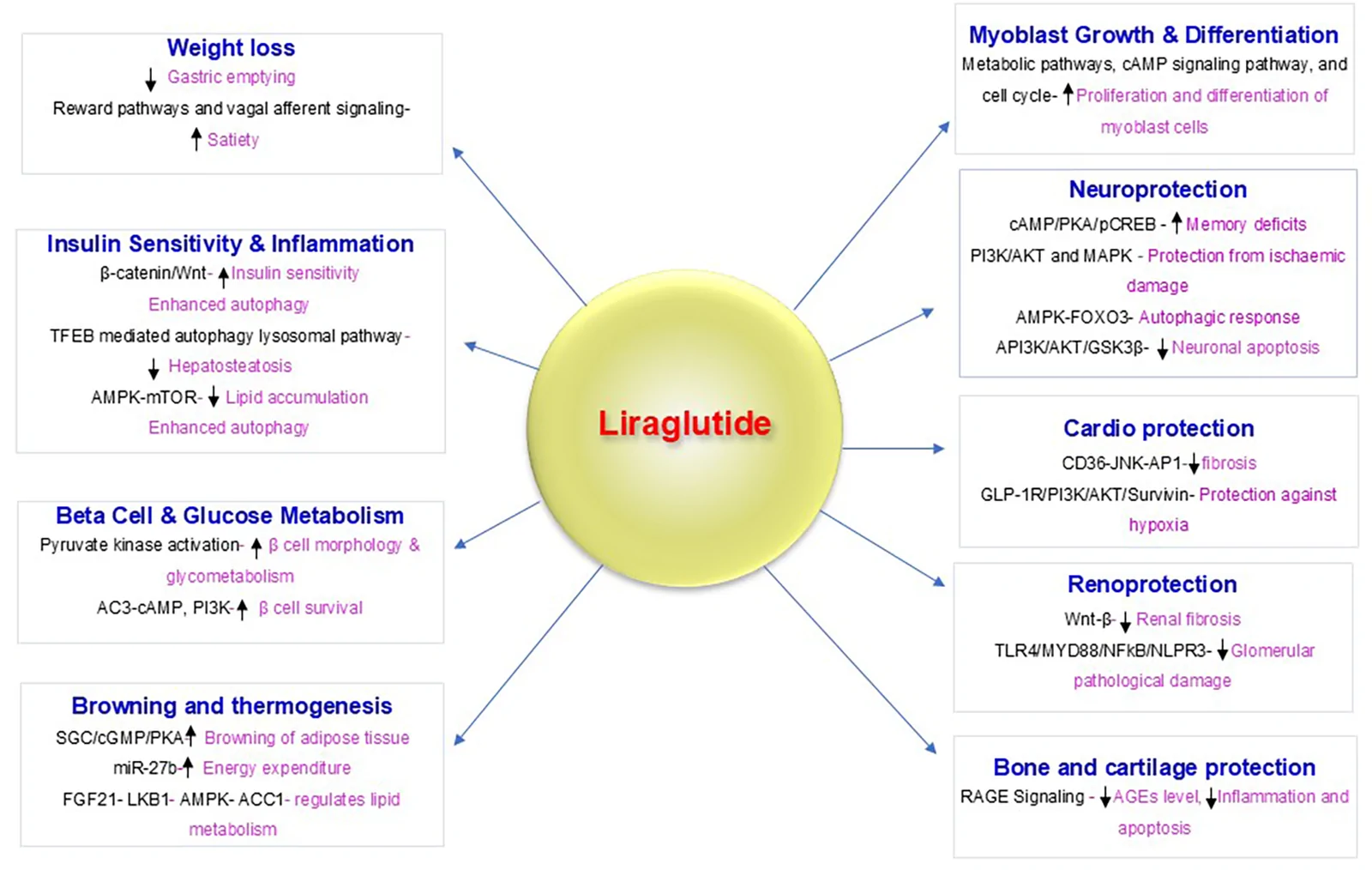
This schematic illustrates the diverse signaling pathway by which liraglutide modulates autophagy, fibrosis, inflammation, apoptosis, lipid accumulation, and cellular morphology & metabolism, exerting the protective effect across multiple organ systems. AC3: Adenylate Cyclase 3; AMPK: AMP-Activated Protein Kinase; AMPK-FOXO3: AMP-Activated Protein Kinase - Forkhead Box O3; AMPK-mTOR: AMP-Activated Protein Kinase - Mechanistic Target of Rapamycin; API3K: Phosphoinositide 3-Kinase; AP1: Activator Protein 1; cAMP: Cyclic Adenosine Monophosphate; cAMP/PKA/pCREB: Cyclic Adenosine Monophosphate/Protein Kinase A/phosphorylated cAMP Response Element-Binding Protein; CD36: Cluster of Differentiation 36; FGF21: Fibroblast Growth Factor 21; GSK3β: Glycogen Synthase Kinase 3 Beta; GLP-1R: Glucagon-Like Peptide-1 Receptor; JNK: c-Jun N-terminal Kinase; LKB1: Liver Kinase B1; MAPK: Mitogen-Activated Protein Kinase; miR-27b: microRNA-27b; mTOR: Mechanistic Target of Rapamycin; MYD88: Myeloid Differentiation Primary Response 88; NFkB: Nuclear Factor kappa-light-chain-enhancer of activated B cells; NLPR3: NOD-, LRR- and pyrin domain-containing protein 3; PKA: Protein Kinase A; PI3K: Phosphoinositide 3-Kinase; pCREB: phosphorylated cAMP Response Element-Binding Protein; RAGE: Receptor for Advanced Glycation End-products; SGC: Soluble Guanylate Cyclase; Survinin: Baculoviral IAP Repeat-Containing Protein 5; TFEB: Transcription Factor EB; TLR4: Toll-Like Receptor 4; Wnt: Wingless/Integrated; β-catenin: Beta-catenin.

Exploring the mechanisms involved in these beneficial clinical outcomes, liraglutide exerts its actions by modulating several key pathways, as summarized in Figure 2, while supplementary Table 1 details the liraglutide- mediated mechanistic orchestration. One strategic way by which liraglutide drives anti-obesity effects is through modulation of fat metabolism and reduction of insulin resistance. Liraglutide upregulates brown fat differentiation, promoting thermogenesis in brown adipose tissue (BAT) via activation of soluble guanylyl cyclase (sGC)/cyclic guanosine phosphate (cGMP)/PKA signalling pathway. ^51^ This increases energy expenditure and heat production mediated by downregulation of miR-27b which facilitates the browning of white adipose tissue. ^52^ Tin addition, liraglutide upregulates adenylate cyclase 3 (AC3) ^53^ at both hepatic and serum levels, indicating a coordinated metabolic regulation.

Liraglutide suppresses gluconeogenic enzymes such as glucose-6-phosphatase and phosphoenolpyruvate carboxykinase via the Wnt/β-catenin pathway, while enhancing expression of glucose transporter type 4 (GLUT4) in both liver and skeletal muscle. ^54^ ^,^ ^55^ It also augments glycolysis and glycogenesis via activation of hexokinase and pyruvate kinase while preserving pancreatic β-cell mass by inhibiting apoptosis through the cAMP-PI3K pathway and increasing proliferation through reducing oxidative stress. ^56^ ^,^ ^57^ It activates autophagy via AMPK and transcription factor EB (TFEB), promoting lysosomal biogenesis and lipid clearance, thus preventing hepatic lipid accumulation. ^58^ ^,^ ^59^ In adipose tissue, it activates fibroblast growth factor 21 (FGF21)/liver kinase B1(LKB1)/AMPK/acetyl-CoA carboxylase 1 (ACC1) axis, thus reducing lipogenesis, promoting fatty acid oxidation, and suppressing pro-inflammatory signalling. ^60^ It also reduces β-site amyloid precursor protein cleaving enzyme 1 (BACE1) activity, thereby exhibiting a neuroprotective action. ^61^ Higher leptin levels observed in both PCOS and obese conditions inhibit follicular growth as well as hinder oocyte maturation. ^62^ Liraglutide in obese mice combats impaired leptin signalling through the Janus kinase 2/signal transducer and activator of transcription 3 (JAK2-STAT3) signalling pathway, lowers expression of improves insulin resistance, lowers fasting insulin levels, decreases hyperandrogenism, and improves menstrual cyclicity pattern, thereby creating a favourable environment relevant to improving reproductive health. ^63^ ^–^ ^68^

Liraglutide demonstrates strong anti-inflammatory activity in PCOS and obese conditions. In human chondrocytes, it inhibits nuclear factor κB (NF-κB)/tumour necrosis factor-alpha (TNF-α) signalling pathway, thereby attenuating extracellular matrix (ECM) proteins degradation and advanced glycation end products (AGE)-induced apoptosis. ^69^ Similarly, in fibroblasts, liraglutide attenuates collagen overproduction by modulating the cluster of differentiation 36 (CD36)/c-Jun N-terminal kinase (JNK)/activator protein 1 (AP1) pathway. ^70^ In addition, it suppresses Wnt/β-catenin and Toll-Like Receptor 4 (TLR4)/myeloid differentiation primary response 88 (MyD88)/NF-κB inflammatory cascade to reduce ECM deposition and pro-inflammatory cytokine expression. ^71^ ^,^ ^72^ It also reduces sclerosis and autophagy in renal cells, ^73^ while in the vasculature, it reduces inflammatory marker expression in obese conditions. ^74^ Further, it enhances bone mineral density and decreases bone resorption, thereby protecting against osteoporosis. ^75^ Its neuroprotective effects have also been reported, with improved neuronal cell survival and repair via cAMP/PKA,CREB and Wnt signalling pathways. ^76^ ^–^ ^79^ In spinal cord and hypoxic ischemic injuries, it preserves tissue integrity by activation of AMPK/Forkhead Box O3 (FOXO3) mediated autophagy and PI3K/Akt/glycogen synthase kinase 3 β (GSK3β) signalling, respectively. ^80^ ^,^ ^81^ It reverses synaptic loss and memory impairment, improves cognition and motor coordination. ^82^ ^–^ ^84^ While these multi-organ effects range from neuroprotection to bone remodelling, highlighting the systemic versatility of liraglutide, they serve as parallel evidence of liraglutide’s ability to conserve and restore cellular homeostasis.

The anti-inflammatory profile of liraglutide is very relevant, and it lowers the level of C-X-C motif chemokine ligand 10 (CXCL10) by blocking the JAK signalling pathway in PCOS condition. ^85^ ^,^ ^86^ It also exhibits potent anti-inflammatory effects by inhibiting the NF-kB and TNF-α pathways, accompanied by upregulation of sirtuin 1 (SIRT1) and increased phosphorylation of insulin receptor substrate (IRS-1). ^87^ ^–^ ^89^ Furthermore, the beneficial effect of liraglutide on decreased inflammation is by lowering key proinflammatory markers, including interleukin-6 (IL-6), interleukin-1beta (IL-1β), and chemokine C-C motif ligand 2 (CCl2). ^90^ Overall, liraglutide highlights the anti-inflammatory effects at both systemic and organ levels, balancing the inflammatory milieu. ^91^ ^–^ ^94^ However, it also warrants further investigation into its effects on ovarian oxidative stress and granulosa cell survival to optimize the use of the drug in correcting reproductive dysfunction in obesity and PCOS conditions.

Liraglutide alters the overall diversity and abundance of gut microbiota, elevates well-known probiotics, ^95^ ^,^ ^96^ promotes bacteria-derived butyrate production and improves lipid profile, inducing weight reduction, regulates glucose metabolism, and reduces insulin resistance and hyperandrogenism, ultimately promoting better metabolic health in obese, PCOS, and diabetic condition. ^97^ ^–^ ^103^ Its effect is mediated by enrichment of the phylum Bacillota, leading to a reduced Bacilliota to Bacteroidota ratio, which is usually lower in the PCOS condition. ^104^ ^–^ ^106^ Clinical trials involving liraglutide have been linked to improved ovarian morphology and function, as well as the restoration of regular menstrual cyclicity. ^68^ These improvements are likely mediated through modulating gut microbiota, which in turn influences hormonal balance and systemic inflammation. However, further clinical trials are warranted to confirm the therapeutic effects of liraglutide.

**Figure 3.**
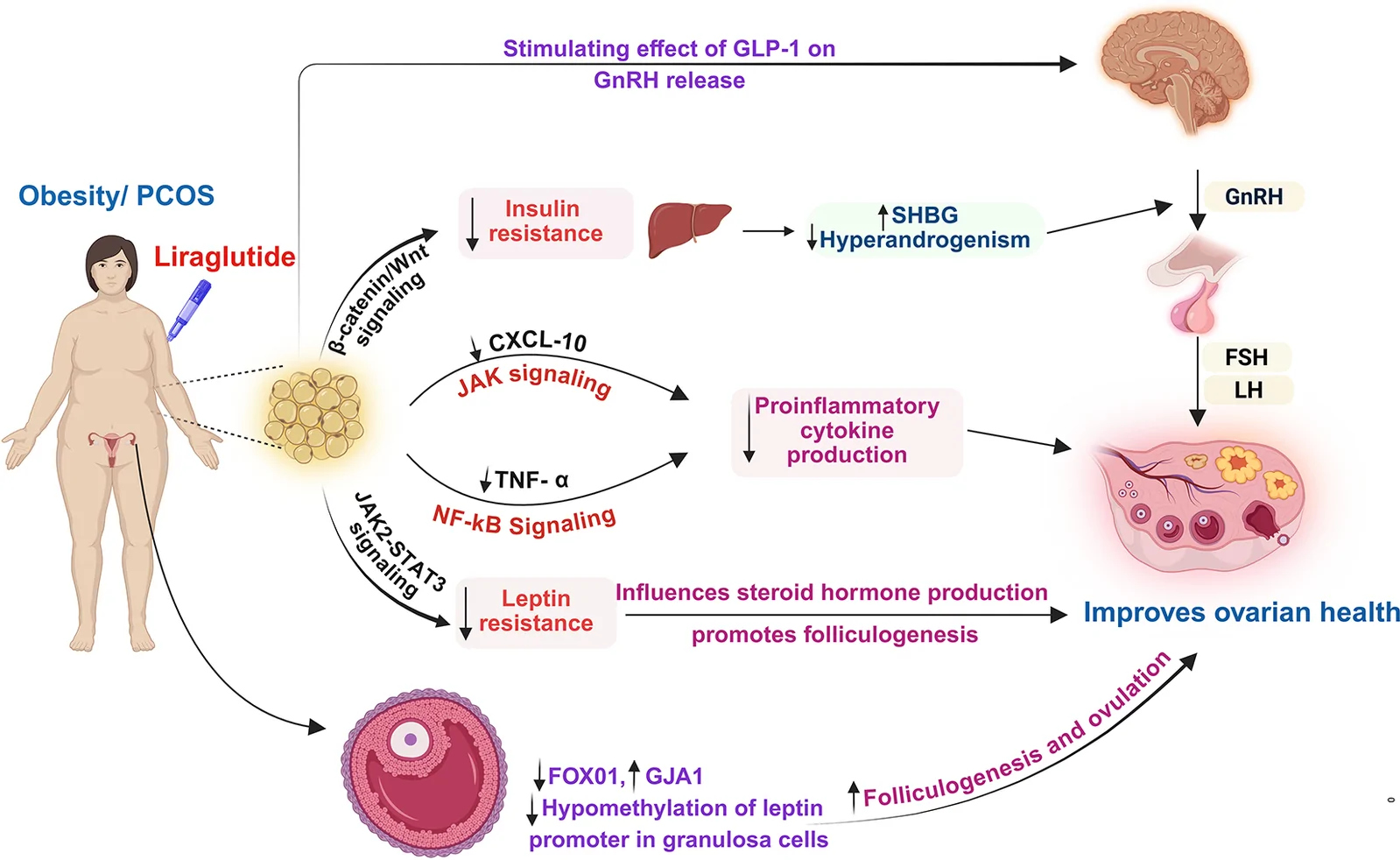
Effect of liraglutide in improving ovarian health explains the mechanism of liraglutide in improving folliculogenesis through various mechanisms (created with BioRender.com). It regulates GnRH pulsatile, FSH and LH level through HPO axis, reduces proinflammatory cytokines, and regulates steroid hormone production thereby affecting ovarian function. At follicle level, It also improves GJA1 protein homeostasis between oocytes and granulosa cells, supporting the effective cellular communication, improves granulosa cell survival by reducing FOXO1 expression, reduces the proinflammatory cytokines in the follicular fluid, reduces the methylation of the leptin promoter in granulosa cells, promoting the improvement in follicular development and ovulation, aiding the improved natural pregnancy rates. (Black upward arrow: increases; black downward arrow: decreases;). CXCL10: C-X-C motif chemokine ligand 10; FSH: Follicle stimulating hormone; FoxO1: Forkhead box protein O1; GJA1: Gap junction protein 1; GnRH: Gonadotropin-releasing hormone; GLP-1: Glucagon-like peptide-1; HPO: Hypothalamus-pituitary-ovary; JAK2: Janus kinase 2; LH: Luteinizing hormone; NF-kB: Nuclear factor kappa-light-chain-enhancer of activated B cells; PCOS: Polycystic ovary syndrome; SHBG: sex hormone-binding globulin; STAT3: signal transducer and activator of transcription 3; TNF-α: Tumor Necrosis Factor alpha; Wnt/β-catenin signaling: Wingless-related integration site/beta-catenin signaling.

One of the promising strategies of liraglutide in improving reproductive health is hormone profile modification by interconnected metabolic and endocrine modifications ( Figure 3). Hyperinsulinemia and elevated LH secretion in obese and PCOS are the key mediators of ovarian-associated hyperandrogenism. Liraglutide reduces adipose tissue inflammation and lipotoxicity and thereby decreases hyperinsulinemia and improves insulin sensitivity, ^107^ which in turn reduces androgen synthesis by following the PI3K pathway mediated CYP17 driven androgen synthesis activity. ^108^ It also improves insulin resistance and elevates SHBG, thereby reducing androgen levels and resulting in a lowered free androgen index. ^109^ ^–^ ^112^ Furthermore, anti-mullerian hormone (AMH) produced by granulosa cells stimulates the pulsatile release of gonadotropin-releasing hormone (GnRH) and downstream gonadotrophins (LH and FSH), which are closely associated with the maintenance of ovarian reserve and function. Notably, AMH levels are often elevated in obese women with PCOS. ^113^ ^,^ ^114^ Marking all the positive alterations in terms of improving the hormonal index, thereby supporting folliculogenesis, liraglutide may be a useful treatment choice for women undergoing PCOS & obesity ( Figure 3).

In addition, liraglutide reduced methylation of the leptin promoter in ovarian granulosa cells in PCOS conditions, which was positively connected to higher ovulation rates, better hormonal profiles, and spontaneous conception. ^115^ However, the translational impact of the findings is constrained by the small sample size and retrospective design, which limits the generalizability. It alters the phosphorylation status of FoxO1, a known negative regulator of cell survival, by promoting Akt-mediated phosphorylation. This leads to reduced expression of pro-apoptotic genes Bim and FasL, decreases apoptosis of granulosa cells, and thereby improves folliculogenesis. ^116^ Liraglutide restores the interaction between granulosa cells and oocytes, which is essential for oocyte growth, by improving the expression of the gap junction protein alpha 1 (GJA1) ^86^ and ferredoxin 1 (FDX1), the latter being critical for p450 enzyme activation in PCOS conditions. ^117^ It also reversed PCOS-induced downregulation of steroidogenic enzymes, namely cholesterol side-chain cleavage enzyme (CYP11A1) and aromatase (CYP19A1) and impaired follicular development. The restoration of these enzymes led to improved estradiol synthesis, folliculogenesis and overall reproductive health. These beneficial effects of liraglutide are further driven by its potential anti-inflammatory effect, which favourably enables the ovarian microenvironment for promoting folliculogenesis and ovulation. Collectively, these beneficial effects position liraglutide as a potential therapeutic option for improving follicular growth in PCOS.

Recent studies have underlined several specific mechanisms through which liraglutide improves ovarian health by supporting follicular development ( Figure 2). Liraglutide reduced methylation of the LEP (leptin) promoter in ovarian granulosa cells in PCOS conditions, ^114^ which positively connected to higher ovulation rates, better hormonal profiles, and spontaneous conception. It alters the phosphorylation status of forkhead box protein O1 (FoxO1), a known negative regulator of cell survival, by promoting Akt-mediated phosphorylation, resulting in reduced expression of pro-apoptotic genes such as Bim and FasL, resulting in decreased apoptosis of granulosa cells and improved folliculogenesis. ^115^ The administration of Liraglutide restores the equilibrium between granulosa cells and oocytes by improving the expression of the gap junction protein alpha 1 (GJA1) in PCOS conditions through the JAK) signaling pathway. ^86^ Importantly, it upregulated Ferredoxin 1 (FDX1) expression in PCOS condition, a protein critical for p450 enzyme activation, which is suppressed in this condition. ^116^ It also reversed PCOS-induced downregulation of steroidogenic enzymes namely cholesterol side-chain cleavage enzyme (CYP11A1) and aromatase (CYP19A1) and impaired follicular development. The restoration of these enzymes led to improved estradiol synthesis, folliculogenesis and overall reproductive health. These beneficial effects liraglutide are further driven by its potential anti-inflammatory effect, which favorably enables the ovarian microenvironment for promoting folliculogenesis and ovulation. The dual function of liraglutide, in increasing granulosa cell survival and reducing inflammation, makes it a potential therapeutic option for improving follicular growth in PCOS conditions.

**Clinical trials assessing the effect of liraglutide on metabolic, hormonal, and reproductive function in obese and PCOS women**

While the mechanistic plausibility of liraglutide in preclinical models is robust, the clinical validation in PCOS/obese population remains a landscape of promise, and available evidence is contingent on methodological heterogeneity. Liraglutide has demonstrated promising effects in improving metabolic and reproductive parameters in non-diabetic women with obesity and PCOS ( Table 2). A randomized, placebo-controlled phase 3 clinical trial involving 82 obese and PCOS patients reported a significant weight reduction in the liraglutide group, compared to the placebo group, leading to regular menstrual cyclicity with two participants achieved spontaneous conception. ^67^ Effects like lowered testosterone, improved insulin resistance, improved lipid profile and adiposity, improved menstrual cyclicity with one participant achieving pregnancy and delivering a healthy child were pronounced in an extreme case of PCOS with endocrine disorder depicted by HAIR-AN syndrome. Interestingly, these benefits occurred with minimal weight loss following post-liraglutide treatment in these patients underscoring its pharmacologic effects independent of weight reduction. ^68^ In a study involving 72 overweight women with PCOS, liraglutide led to reduction in body weight. Increase in SHBG level and decrease in free testosterone level. Although it did not improve insulin sensitivity, reductions in fasting glucose and leptin levels were observed. This study did not elaborate on reproductive functions as well as hormone levels. ^109^ Furthermore, in a cohort of 30 obese PCOS patients, liraglutide was superior to metformin in improving glucose metabolism, lipid profiles, and BMI, as well as reducing leptin promoter methylation in granulosa cells, thereby increasing leptin secretion in these cells. Moreover, significant reductions in FSH, LH, and E2 levels were observed after liraglutide intervention, alongside improved menstrual regularity, ovulation rates, and natural pregnancy occurrences. ^110^ However, this study lacked a placebo group, had a small sample size. In another study involving obese women with PCOS, liraglutide treatment resulted in greatest weight loss, followed by roflumilast and metformin, with metformin showing the least among the three treatment groups. All three treatment groups experienced improvements in menstrual regularity. However, roflumilast had a significant upper hand over the liraglutide arm. No significant intergroup differences in free testosterone, SHBG, androstenedione, dehydroepiandrosterone (DHEA), FSH, and LH were observed. In addition, improved glucose homeostasis was found in liraglutide patients compared to metformin and roflumilast subjects. However, this study had a few limitations, including a small sample size, short duration, open-label nature of the study, and lack of active lifestyle promotion. ^117^ In contrast, placebo-controlled, randomized trial involving 65 overweight PCOS women treated with liraglutide for 26 weeks showed average weight loss, improved fasting glucose, improved insulin sensitivity, menstrual regularity and normalization of AMH level. Further, three-dimensional ultrasound revealed a decrease in both total ovarian volume and stromal volume. However, this study also had a limitation of participant selection bias, as partial recruitment was by social media, and lifestyle changes were not actively promoted throughout the study. ^118^ Among the Chinese population, the combination therapy of metformin and liraglutide has proved its efficacy in weight loss and improving hyperandrogenemia, including total testosterone, free androgen index, and SHBG levels and showed a menstrual cycle recovery rate of 92.59%, whereas the metformin alone group showed 88% recovery rate. ^110^ Nevertheless, limitations included the absence of a control group, potential selection bias, lack of fertility outcome data, short follow-up duration, and their small sample size, single-center design. In addition, this study did not assess key reproductive parameters such as ovulation or pregnancy outcomes.

**Table 2.** Clinical evidence of liraglutide alone or in combination with metformin improving in reproductive, metabolic, and endocrine dysfunction in obese and PCOS women.

Author, study & design	Population	Dosage & treatment duration of liraglutide	Effect of liraglutide on metabolic, endocrine, reproductive changes
Elkind-Hirsch et al. ^67^ Double-blind RCT	Obese, PCOS women	Liraglutide 3 mg/day vs. placebo + lifestyle, for 32 weeks.	Decreased weight, and increased insulin sensitivity. Improved endocrine and metabolic markers over placebo. More regular menstrual cyclicity and out of 44 patients, 2 pregnancies, healthy births.
Livadas et al. ^68^ Case Study	HAIR-AN syndrome/PCOS women	Liraglutide 1.8 mg/day, for 14 months.	Decrease of insulin resistance, androgen levels, with improvement of fat deposition. More regular menstrual cyclicity and out of 5 patients, 1 pregnancy, live birth.
Jensterle et al. ^117^ Prospective RCT	Obese, PCOS women	Liraglutide 1.2 mg QD, s.c. vs. metformin 1000 mg BID or roflumilast 500 μg, QD, 12 weeks.	No significant change in hyperandrogenism. Increased menstrual frequency outperformed metformin in reproductive parameters.
Nylander et al. ^118^ Double-blind RCT	Overweight PCOS women	Liraglutide 1.8 mg vs. placebo, 26 weeks follow-up.	Effective weight loss, decreased fasting glucose, hemoglobin A1c, increased SHBG, and decreased free testosterone. Decreased ovarian and stromal volume, and more regular menstrual cyclicity.
Xing et al. ^110^ Prospective, Open-label RCT	PCOS women	Metformin 1000 mg BID or liraglutide 1.2 mg QD, s.c. + metformin for 12 weeks.	In combination with metformin, it improved bodyweight, fasting glucose, hyperandrogenism, and sex hormone balance. Recovery rate of the menstrual cycle was better than metformin group in the combination group.
Jensterle et al. ^121^ Open-label RCT	Obese, PCOS women	Liraglutide 1.2 mg QD vs. metformin + liraglutide for 12 weeks.	In combination with metformin Increased glucose metabolism, decreased BMI, decreased androstenedione, and greater improvements in ovarian morphology and metabolism.
Long et al. ^122^ Retrospective	Obese, PCOS women	Liraglutide 0.6 mg QD + metformin 0.85 g BID for 12 weeks.	In combination with metformin, it improved lipid metabolism, insulin sensitivity, decreased free androgen index better then when metformin given alone. Reproductive functions not assessed.
Salamun et al. ^123^	Prospective RCT Infertile, obese PCOS women	Metformin1000 mg BID vs. metformin + liraglutide 1.2 mg QD for 12 weeks.	In combination with metformin, it decreased fasting glucose, OGTT, HOMA-IR, decreased LH & testosterone, improved metabolic and endocrine outcomes. Increased pregnancy rate per embryo transfer (85.7% in combination with metformin).

BID: Bis in die (twice daily); BMI: Body Mass Index; HAIR-AN: Hyperandrogenism, Insulin Resistance, and Acanthosis Nigricans; HbA1c: Hemoglobin A1c; HOMA-IR: Homeostatic Model Assessment of Insulin Resistance; LH: Luteinizing Hormone; OGTT: Oral Glucose Tolerance Test; PCOS: Polycystic Ovary Syndrome; QD: Quaque die (once daily); RCT: Randomized Controlled Trial; s.c.: Subcutaneous; SHBG: Sex Hormone-Binding Globulin.

Adding on, the combination of liraglutide with metformin, appears to offer synergistic benefits on metabolic and reproductive outcomes in obese women with PCOS. Combination of liraglutide and metformin not only offers superior glycemic control more effectivly but also provides a significant advantage in promoting body weight loss, emphasizing its role in metabolic regulation in comparison to placebo, sitagliptin, glimepiride, dulaglutide, insulin glargine, and NPH insulin. ^119^ ^,^ ^120^ Importantly, the combined treatment did not increase the risk of hypoglycemia but induced a higher incidence of adverse effects of gastrointestinal disturbances, such as nausea, compared to metformin. However, the severity of these adverse effects was reduced over time. ^121^ Nevertheless, limitations included the absence of a control group, potential selection bias, lack of fertility outcome data, short follow-up duration and their small sample size, single-center design. ^122^ Few studies did not assess key reproductive parameters such as ovulation, menstrual cyclicity, or pregnancy outcomes. Subsequently infertile obese PCOS patients treated either with metformin in combination with low-dose liraglutide, resulting in markedly higher pregnancy rates per embryo transfer in the liraglutide despite similar reductions in weight and visceral adiposity. ^123^ Higher cumulative pregnancy rates are supported by improved menstrual cycle regularity, glucose metabolism, and modulation of LH and progesterone levels. These findings suggest liraglutide or in combination with metfromin is effective for weight reduction and regulated hormonal balance in women with obesity and PCOS, which supports its potential as a therapeutic option that may exert beneficial effects on reproductive outcomes in women with metabolic disorders, providing a potential therapeutic option for addressing infertility in this population.

## Discussion

Liraglutide, the foremost GLP-1 R agonist, has emerged as pivotal in the dual management of obesity and diabetes. It seeks more attention due to its rise in global consumption, potentially enabling a more holistic and comprehensive treatment approach. Notably, along with clinical triumphs of successful weight loss and adipokine rebalancing, liraglutide reshapes its therapeutic horizon, directing towards synced treatment for reproductive dysfunction. Liraglutide has demonstrated its multifaceted effects in the PCOS murine model, indicating the rescue of folliculogenesis, ^86^ improvement of granulosa cell proliferation, ^114^ which are crucial for balanced steroidogenesis, and alleviation of a proinflammatory environment. Crucially, clinical trials have reinforced the effectiveness of the drug, which significantly improved both pregnancy rates in combination with metformin and successful embryo transfer. ^124^ Comparatively, metformin has long served as the most widely used pharmacological therapy in PCOS due to its insulin-sensitizing effects, affordability, and extensive clinical experience. In contrast, liraglutide promotes weight reduction primarily through appetite regulation, delayed gastric emptying, and enhanced satiety signaling. As a result, liraglutide often produces greater weight loss and improvements in metabolic parameters compared with metformin monotherapy. These differences suggest that GLP-1 receptor agonists may occupy an important position as adjunctive therapy, particularly in individuals who do not achieve adequate metabolic control with lifestyle modification and insulin sensitizers alone. However, a dose-dependent weight loss was observed in subjects receiving liraglutide, which was found to be better than orlistat. While these results are promising, transient GI effects such as nausea, vomiting, headache, diarrhoea, constipation, and injection site reactions, and the non-occurrence of pregnancy led to 18% dropout rate among the patients. The other limitations included were short trial duration, lack of ovulation or live birth assessment, and absence of clinical evaluation of hirsutism. ^41^ ^,^ ^67^ However, no events of acute pancreatitis were observed, as seen in other GLP-1 Ras. In addition to these, the other limitations noted were the absence of a control group, potential selection bias, short follow-up duration, small sample size, single-centre design and did not assess key reproductive parameters such as ovulation or pregnancy outcomes. ^110^ ^,^ ^118^ Beyond these short-term adverse effects, the reproductive safety of liraglutide is questionable and hence recommended to discontinue before planned conception, due to limited human data regarding pregnancy outcomes.

Current evidence suggests favourable metabolic effects; the long-term endocrine consequences of the sustained effect of liraglutide in reproductive-age women remain incompletely understood. Continued pharmacovigilance and longitudinal studies are therefore necessary to clarify these aspects. Liraglutide encapsulated tannic acid and aluminium ion nanoparticles have proved durable and painless with long-term therapeutic efficacy. Efforts in this direction have further led to exploring the oral route of intake. Prolonged release of liraglutide by chitosan nanoparticles, and protection of liraglutide from degradation by gastric and intestinal fluid has shed a ray of hope. ^125^ ^–^ ^128^ The right mode of administration also may reduce the side effects, like gastrointestinal reactions, traditionally observed in liraglutide dosage. ^129^ ^,^ ^130^ Further studies are required in the direction of providing an affordable generic version of liraglutide, evaluating the long-term durability of the reproductive benefit, and identifying biomarkers that could also pave the way for personalised therapy in improving fertility, as well as tailored dosage.

## Conclusion

Liraglutide is a crucial addition to the therapeutic options for women with PCOS-related metabolic and reproductive dysfunction. Evidence from randomized controlled trials and retrospective studies highlights the efficacy of liraglutide in modulating hormone levels and improving insulin sensitivity, which may contribute to regular menstrual cyclicity. Notably, a limited number of studies have further investigated liraglutide’s direct impact on reproductive success by assessing and demonstrating increased natural pregnancy rates and responses to ART. However, these findings remain preliminary. Despite these successes and advancements, critical limitations to consider include brief intervention durations, small sample sizes, lack of ovulation tracking, cost of treatment and limited longitudinal follow-up of live birth data. Furthermore, important considerations such as treatment accessibility and uncertainties regarding reproductive safety must be carefully weighed when considering liraglutide for women of reproductive age. Future research should focus on exploring the biochemical mechanisms underlying liraglutide’s long-term effects on reproductive health through improvements in weight and insulin sensitivity, rather than as a definitive fertility therapy. By embracing cutting-edge treatments like liraglutide, we can better address the complex challenges of infertility and empower women on their journey to reproductive health. Additionally, further research in this area will enhance the lives of women facing these challenges and lead to improved clinical outcomes. The findings underscore the importance of individualised treatment strategies in addressing the intricate issues of PCOS and obesity-related infertility. At present, liraglutide should be viewed primarily as a metabolic therapy that may indirectly support reproductive health through improvements in weight and insulin sensitivity rather than as a definitive fertility treatment.

## Ethical approval

Ethical approval and consent were not required.

## Data Availability

The data availability statement for this study has been duly registered and archived in the Biostudies repository, which is recognized for its commitment to the accessibility and preservation of scientific data. The extended data ( **Supplementary Table 1)** supported by this study is publicly available and can be accessed at the following DOI link: https://doi.org/10.6019/S-BSST2135. ^131^ Data are available under the terms of the CC0 1.0 Universal.
