# Supplementary material for: Role of Liraglutide in weight management and reproductive health in women with obesity and PCOS- A critical review of the evidence
Source: F1000Res. 2026 Apr 24;14:979. Originally published 2025 Sep 24. [Version 2] doi: 10.12688/f1000research.167998.2 (PMC13555055; doi:10.12688/f1000research.167998.2)
Supplement: Peer review history

# Reviewer report: Reviewer response for version 2

**DOI:** 10.5256/f1000research.197883.r499322

**Published:** 2026-07-27

**Authors:** Vikas Dighe

**Affiliations:** 1 ICMR-National Institute for Research in Reproductive and Child Health, Mumbai, India

The author has discussed the role of liraglutide in the management of obesity and polycystic ovary syndrome (PCOS). The comparative effectiveness of liraglutide with other GLP-1 receptor agonists in improving weight loss and glycaemic control has been clearly highlighted. Furthermore, the manuscript effectively explains the impact of liraglutide on multiple aspects of PCOS pathophysiology, including metabolic, endocrine, inflammatory, hyperinsulinemic, and reproductive functions.

 Given that PCOS is a multifactorial disorder involving metabolic, endocrine, reproductive, inflammatory, steroidogenesis, folliculogenesis, and gut microbiota-related alterations, the author has mm,outlined the pathways through which liraglutide exerts its beneficial effects. This supports its potential as a promising therapeutic option for PCOS management. Additionally, the inclusion of clinical trial data, although derived from a small cohort, along with a discussion of treatment limitations, strengthens the manuscript.

 The author has also appropriately highlighted the drawbacks and limitations of certain studies, particularly those that did not assess key reproductive outcomes such as fertility, ovulation, pregnancy outcomes, and live birth rates.

 However, the author is advised to revise the terminology by referring to PCOS as PMOS (Polyendocrine Metabolic Ovarian Syndrome) consistently throughout the manuscript.

Is the review written in accessible language?

Yes

Are all factual statements correct and adequately supported by citations?

Yes

Are the conclusions drawn appropriate in the context of the current research literature?

Yes

Is the topic of the review discussed comprehensively in the context of the current literature?

Yes

Reviewer Expertise:

Endocrine disorders

I confirm that I have read this submission and believe that I have an appropriate level of expertise to confirm that it is of an acceptable scientific standard.

# Reviewer report: Reviewer response for version 2

**DOI:** 10.5256/f1000research.197883.r478732

**Published:** 2026-05-23

**Authors:** Dr. Abilash V G

**Affiliations:** 1 Vellore Institute of Technology (VIT), Vellore, Tamilnadu, India

The authors have thoroughly revised the manuscript as per the comments. The final version of the revised manuscript can be accepted for indexing.

Is the review written in accessible language?

Yes

Are all factual statements correct and adequately supported by citations?

Partly

Are the conclusions drawn appropriate in the context of the current research literature?

Partly

Is the topic of the review discussed comprehensively in the context of the current literature?

Partly

Reviewer Expertise:

Reproductive Biology, Biomedical Genetics

I confirm that I have read this submission and believe that I have an appropriate level of expertise to confirm that it is of an acceptable scientific standard.

# Response

**Published:** 2026-05-23

**Authors:** Sandhya Kumari M V

**Affiliations:** Reproductive Science, Not Applicable, Manipal, Karnataka, India

Dear Dr. Abilash V G,

 We sincerely thank you for the careful review of our revised manuscript. We greatly appreciate the constructive comments and valuable suggestions you provided during the review process, which helped improve the quality and clarity of our manuscript. We are grateful for your approval and thoughtful consideration of our work.

# Reviewer report: Reviewer response for version 1

**DOI:** 10.5256/f1000research.185150.r455513

**Published:** 2026-02-25

**Authors:** Dr. Abilash V G

**Affiliations:** 1 Vellore Institute of Technology (VIT), Vellore, Tamilnadu, India

The manuscript entitled *“Role of Liraglutide in weight management and reproductive health in women with obesity and PCOS -A critical review of the evidence”* addresses an important and clinically relevant topic at the intersection of metabolic endocrinology and reproductive medicine. The growing interest in GLP-1 receptor agonists as potential adjuncts in PCOS management makes this review timely. The authors provide a broad overview of mechanistic pathways and summarize clinical observations linking liraglutide to metabolic and reproductive outcomes. However, in its present form, the manuscript requires substantial revision before it can meet the standards expected for indexing. The principal concerns relate to methodological transparency, depth of critical synthesis, conceptual focus, and interpretive balance.

- Although multiple databases and general keywords are reported, the search strategy is not described in a manner that allows replication. There is no indication of structured screening procedures, eligibility assessment, or study selection workflow, nor is there any assessment of bias or study quality.
- The inclusion criteria appear subjective, and the absence of defined temporal limits further weakens methodological clarity. Even if the intention is to present a narrative review rather than a systematic one, greater transparency regarding literature identification and selection is essential. A structured reporting framework, such as a PRISMA-style flow description or an equivalent narrative justification, would significantly strengthen the credibility of the work.
- A more fundamental issue is that the manuscript describes itself as a critical review, yet the tone and structure remain largely descriptive. Much of the literature is summarized without rigorous appraisal of study design, limitations, or evidentiary strength. Positive findings are frequently highlighted without proportionate discussion of methodological weaknesses or conflicting data. A true critical review should interrogate heterogeneity across studies, address confounding factors, and clearly distinguish between mechanistic plausibility, preclinical inference, and clinically validated outcomes.
- Several sections extend extensively into areas only tangentially related to reproductive outcomes, including neuroprotection, bone metabolism, and systemic inflammatory modulation across multiple organ systems. Although these discussions demonstrate breadth of knowledge, they detract from the central focus and dilute the narrative cohesion of the review. The manuscript would benefit from tighter thematic discipline, prioritizing content directly relevant to reproductive endocrinology and PCOS-associated infertility, while condensing or relocating peripheral material.
- Another limitation lies in insufficient integration across thematic sections. Mechanistic pathways, microbiome modulation, hormonal regulation, and clinical outcomes are treated largely in isolation. The manuscript would be substantially improved by synthesizing these strands into a unified conceptual model linking molecular action to clinical translation. Such integration would help readers understand how mechanistic insights support or fail to support therapeutic outcomes, thereby strengthening the translational relevance of the review.
- The discussion of comparative therapies is also underdeveloped. While metformin and other anti-obesity pharmacotherapies are mentioned, there is little analytical comparison regarding efficacy, safety, accessibility, or positioning within treatment algorithms. Given that clinical decision-making involves evaluating alternatives rather than isolated interventions, a comparative framework is essential. Similarly, the discussion of safety remains relatively superficial. Adverse effects, cost barriers, and administration challenges are noted, but the manuscript does not explore reproductive safety, pregnancy outcomes, or potential long-term endocrine implications in sufficient depth.
- The conclusion section adopts a tone that appears more optimistic than warranted by the evidence presented. The manuscript itself acknowledges limitations in existing research, including short follow-up durations and incomplete reproductive outcome assessment.
- The concluding statements should more carefully reflect these uncertainties and emphasize the provisional nature of current findings. A more balanced summary would enhance the credibility and scholarly neutrality of the work.

Is the review written in accessible language?

Yes

Are all factual statements correct and adequately supported by citations?

Partly

Are the conclusions drawn appropriate in the context of the current research literature?

Partly

Is the topic of the review discussed comprehensively in the context of the current literature?

Partly

Reviewer Expertise:

Reproductive Biology, Biomedical Genetics

I confirm that I have read this submission and believe that I have an appropriate level of expertise to confirm that it is of an acceptable scientific standard, however I have significant reservations, as outlined above.

# Response

**Published:** 2026-03-18

**Authors:** Sandhya Kumari M V

**Affiliations:** Reproductive Science, Not Applicable, Manipal, Karnataka, India

**Author's response to Reviewer comments:**

 The manuscript entitled *“Role of Liraglutide in weight management and reproductive health in women with obesity and PCOS -A critical review of the evidence”* addresses an important and clinically relevant topic at the intersection of metabolic endocrinology and reproductive medicine. The growing interest in GLP-1 receptor agonists as potential adjuncts in PCOS management makes this review timely. The authors provide a broad overview of mechanistic pathways and summarize clinical observations linking liraglutide to metabolic and reproductive outcomes. However, in its present form, the manuscript requires substantial revision before it can meet the standards expected for indexing. The principal concerns relate to methodological transparency, depth of critical synthesis, conceptual focus, and interpretive balance.

***We would like to express our gratitude to the Reviewer for their critical assessment of this manuscript. We are pleased that the Reviewer found the research both timely and clinically relevant. Below, we provide comprehensive responses to each point raised and hope the revised version will now meet the criteria for publication.***

 1. Although multiple databases and general keywords are reported, the search strategy is not described in a manner that allows replication. There is no indication of structured screening procedures, eligibility assessment, or study selection workflow, nor is there any assessment of bias or study quality.

***Response: We thank the reviewer for this critical assessment. We acknowledge that the initial description did not sufficiently detail the selection workflow or the criteria for evidence evaluation required for a reproducible study. In response, we have revised the methodology of the manuscript to clarify that this work is a narrative review. which intends to provide a comprehensive synthesis of the complex 'mechanistic orchestra' between liraglutide, obesity, and PCOS. To enhance methodological transparency, we have updated the 'Methods' section to include a structured, hierarchical selection workflow, defined temporal limits (inception to March 2025), and a clear rationale for the inclusion of the articles that form the core of our translational analysis. We have updated the methodology to state that, to minimize the bias, the literature survey including the title, abstract and the full text screening, was conducted independently by two researchers.***

 2. The inclusion criteria appear subjective, and the absence of defined temporal limits further weakens methodological clarity. Even if the intention is to present a narrative review rather than a systematic one, greater transparency regarding literature identification and selection is essential. A structured reporting framework, such as a PRISMA-style flow description or an equivalent narrative justification, would significantly strengthen the credibility of the work.

***Response: We thank the reviewer for the constructive criticism regarding the transparency of the literature selection process. We recognise that a more formalised approach is essential for the credibility of the review. To address this, we have implemented the following revisions. (1) Temporal limits are re-specified to give methodological clarity. (2) We have described the narrative justification of the workflow, and PRISMA-style flow description, as the manuscript is a narrative review. This will ensure the credibility and replicability of the literature search of the work.***

 3. A more fundamental issue is that the manuscript describes itself as a critical review, yet the tone and structure remain largely descriptive. Much of the literature is summarized without rigorous appraisal of study design, limitations, or evidentiary strength. Positive findings are frequently highlighted without proportionate discussion of methodological weaknesses or conflicting data. A true critical review should interrogate heterogeneity across studies, address confounding factors, and clearly distinguish between mechanistic plausibility, preclinical inference, and clinically validated outcomes.

***Response: We thank the reviewer for the pivotal critique. We recognise that the initial draft leaned more towards the descriptive summary, with the mention of limitations. We have revised the ‘’Clinical trials assessing the effect of liraglutide on metabolic, hormonal, and reproductive function in obese and PCOS women’’ section to align with the analytical framework as suggested by explicitly discussing heterogeneity and confounding factors. We have restructured the discussion to clearly distinguish between preclinical mechanistic pathways and clinically validated outcomes clearly. This ensures that the potential of Liraglutide is framed as plausible based on animal data, rather than proven in clinical practice.***

 4. Several sections extend extensively into areas only tangentially related to reproductive outcomes, including neuroprotection, bone metabolism, and systemic inflammatory modulation across multiple organ systems. Although these discussions demonstrate breadth of knowledge, they detract from the central focus and dilute the narrative cohesion of the review. The manuscript would benefit from tighter thematic discipline, prioritizing content directly relevant to reproductive endocrinology and PCOS-associated infertility, while condensing or relocating peripheral material.

***Response: We value the reviewer’s insight regarding the narrative cohesion of the review. We recognise that while liraglutide has a broad systemic effect, the extensive detailing of the same may have diluted the primary focus of the article. We address this by significantly condensing the discussion of the peripheral systemic effects.***

 5. Another limitation lies in insufficient integration across thematic sections. Mechanistic pathways, microbiome modulation, hormonal regulation, and clinical outcomes are treated largely in isolation. The manuscript would be substantially improved by synthesizing these strands into a unified conceptual model linking molecular action to clinical translation. Such integration would help readers understand how mechanistic insights support or fail to support therapeutic outcomes, thereby strengthening the translational relevance of the review.

***Response: We thank the reviewer for this valuable observation. In response, we have revised the manuscript to better integrate the thematic sections by explicitly highlighting the interconnected nature of mechanistic pathways, microbiome modulation, hormonal regulation, and clinical outcomes. Rather than presenting these elements in isolation, the revised text emphasizes how these processes interact within a complex biological system and collectively contribute to the therapeutic effects of liraglutide. This integrated approach aims to strengthen the translational relevance of the review by linking molecular mechanisms with clinical outcomes more coherently.***

 6. The discussion of comparative therapies is also underdeveloped. While metformin and other anti-obesity pharmacotherapies are mentioned, there is little analytical comparison regarding efficacy, safety, accessibility, or positioning within treatment algorithms. Given that clinical decision-making involves evaluating alternatives rather than isolated interventions, a comparative framework is essential. Similarly, the discussion of safety remains relatively superficial. Adverse effects, cost barriers, and administration challenges are noted, but the manuscript does not explore reproductive safety, pregnancy outcomes, or potential long-term endocrine implications in sufficient depth.

***Response: We thank the reviewer for this critical feedback. We have fundamentally restructured the discussion to provide a comparative framework and insights into clinical safety. To ensure the conceptual focus of the manuscript remains centred on established clinical practice and translational relevance, and the primary focus of this review is the potential of liraglutide within the reproductive axis, we have included a comparative analysis with existing standard of care therapies***

 7. The conclusion section adopts a tone that appears more optimistic than warranted by the evidence presented. The manuscript itself acknowledges limitations in existing research, including short follow-up durations and incomplete reproductive outcome assessment.

***Response: We thank the reviewer for this critical analysis. We acknowledge that the initial conclusion may have understated the clinical uncertainties presented in the body of the article. We have revised the conclusion to adapt more tempered and balanced tone. Specifically, we have acknowledged the limitations such as brevity of trial durations of drug administration and follow-up and a major lack of longitudinal live-birth data and present it more carefully.***

 8. The concluding statements should more carefully reflect these uncertainties and emphasise the provisional nature of current findings. A more balanced summary would enhance the credibility and scholarly neutrality of the work.

***Response: We acknowledge the reviewer's point regarding the need for a more balanced and neutral scholarly tone. We have substantially revised the conclusion to ensure that our final statement now explicitly weighs the robust mechanistic promise existing in preclinical models as well as clinical uncertainty. Detailed limitations and future directions are addressed in the Discussion to avoid redundancy, while the Conclusion has been streamlined to provide a concise and neutral summary.***
